# Stimuli-Responsive Nanozyme Reprograms Tumor Immunometabolism and Overcomes Therapeutic Resistance in Hepatocellular Carcinoma

**DOI:** 10.1021/acsnano.5c11352

**Published:** 2026-01-06

**Authors:** Yen-Nhi Ngoc Ta, Van-Anh Thi Nguyen, Thu-Thuy Can, Meng-Cheng Hsieh, Bang Giang Thi Cao, Dehui Wan, Chian-Hui Lai, Chun-Chieh Wu, Fu-Fei Hsu, Yu-Ting Yen, Shen-Nien Wang, Yunching Chen

**Affiliations:** † Institute of Biomedical Engineering, 34881 National Tsing Hua University, Hsinchu 30013, Taiwan; ‡ International Intercollegiate PhD Program, 34881 National Tsing Hua University, Hsinchu 30013, Taiwan; § Graduate Institute of Biomedical Engineering, 34916 National Chung Hsing University, Taichung 40227, Taiwan; ∥ Department of Pathology, 89234 Kaohsiung Medical University Hospital, Kaohsiung 80708, Taiwan; ⊥ Institute of Biomedical Sciences, 38017 Academia Sinica, Taipei 11529, Taiwan; # Institute of Translational Medicine and New Drug Development, School of Medicine, China Medical University, Taichung 40402, Taiwan; ∇ College of Medicine, 38023 Kaohsiung Medical University, Kaohsiung 80708, Taiwan; ○ Pingtung Hospital, Ministry of Health and Welfare, Pingtung 900, Taiwan; ◆ Department of Chemistry, 34881 National Tsing Hua University, Hsinchu 30013, Taiwan

**Keywords:** hepatocellular carcinoma (HCC), glucose oxidase (GOx), immunometabolism, glycolysis, immunotherapy, pH-responsive enzyme delivery

## Abstract

Hepatocellular carcinoma (HCC) exhibits profound glycolytic reprogramming that drives tumor growth, impairs apoptosis, and suppresses immune responses, leading to resistance against conventional therapies. To overcome this challenge, we developed a stimuli-responsive nanozyme composed of a pH-sensitive lipid–gelatin–protamine (LGP) nanogel encapsulating glucose oxidase (GOx). This tumor-selective nanozyme depletes intratumoral glucose under acidic conditions, inducing oxidative and endoplasmic reticulum stress, upregulating death receptors, and sensitizing HCC cells to TRAIL- and doxorubicin (DOX)-induced apoptosis. Co-delivery of GOx and DOX within the nanozyme reprograms tumor immunometabolism, enhancing immunogenic cell death and promoting the release of damage-associated molecular patterns (DAMPs). These changes stimulate dendritic cell maturation and cytotoxic CD8^+^ T-cell activation. Transcriptomic profiling confirms that this nanozyme remodels the immunosuppressive microenvironment by suppressing metabolic pathways while activating immune-related gene programs. When combined with an anti–PD-1 checkpoint blockade, the nanozyme elicits potent tumor regression and abrogates metastasis without systemic toxicity in orthotopic HCC models. Overall, this work introduces a multifunctional tumor-responsive nanozyme that integrates metabolic intervention, apoptotic priming, and immune activation to overcome therapeutic resistance in the HCC.

## Graphical abstract

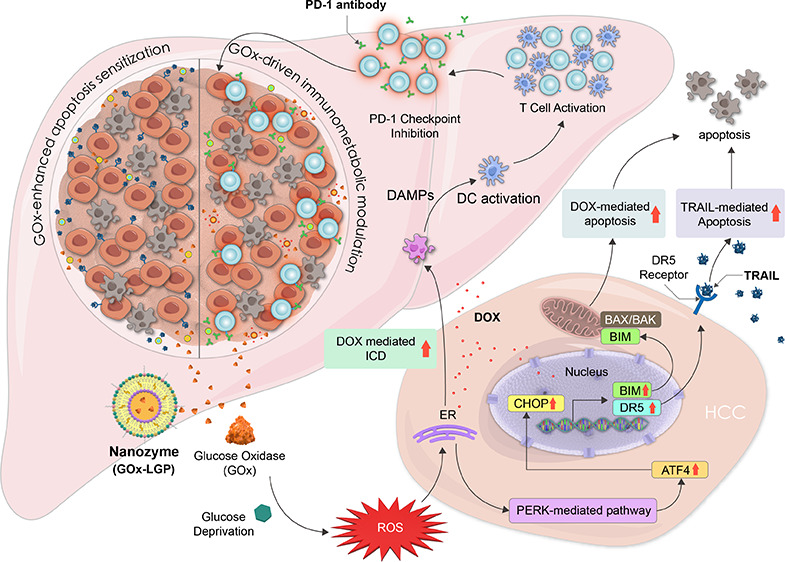

## 1 Introduction

Hepatocellular carcinoma (HCC) is an aggressive primary liver cancer and a leading cause of cancer-related mortality worldwide.^1^ Despite the availability of chemotherapeutics, tyrosine kinase inhibitors, and immune checkpoint inhibitors, systemic treatments offer limited and often short-lived benefits due to intrinsic and adaptive resistance.^2^ This highlights the urgent need for therapeutic strategies that can overcome drug resistance and achieve more durable tumor control. One of the major drivers of drug resistance in HCC is metabolic reprogramming. HCC cells exhibit a Warburg-like phenotype, relying on aerobic glycolysis to fuel rapid growth and survival.^3^ Upregulation of glucose transporters and glycolytic enzymes enables excessive glucose uptake and lactate production, promoting proliferation, evasion of apoptosis, and metastatic potential.^4–6^ High glycolytic activity also enhances antioxidant defenses, limiting oxidative stress and conferring resistance to redox-mediated and chemotherapeutic apoptosis.^7^ Concurrently, lactate accumulation and acidic byproducts suppress cytotoxic T- and NK-cell function and foster an immunosuppressive tumor microenvironment, forming an immunometabolic barrier that limits immunotherapy efficacy.^8,9^ Thus, glycolysis not only sustains tumor progression but also reinforces resistance to apoptosis and immune attack.^10^

Targeting glycolysis has emerged as a promising therapeutic strategy to exploit the metabolic dependencies of cancer cells. Numerous preclinical studies have explored small-molecule inhibitors targeting key glycolytic enzymes, such as 2-deoxy-d-glucose (2-DG) targeting hexokinase II, FX11 inhibiting lactate dehydrogenase A, and dichloroacetate (DCA) modulating pyruvate dehydrogenase kinase.^11–13^ While these agents disrupt glycolytic flux and reduce ATP production, their therapeutic potential is often constrained by systemic toxicity, limited tumor selectivity, and metabolic compensation.^14^ To address these challenges, enzyme-based glucose depletion strategies have gained attention, offering more direct and sustained suppression of glycolysis.^15,16^ In particular, glucose oxidase (GOx) offers unique advantages due to its dual catalytic function—consuming glucose and producing hydrogen peroxide—thereby inducing both metabolic stress and oxidative damage.^17–19^ Thus, GOx not only disrupts energy metabolism but also induces immunogenic cell death, thereby reshaping the tumor microenvironment toward a more immunostimulatory state. Compared with conventional inhibitors, GOx exerts stronger metabolic pressure and has shown greater potential to sensitize tumors to both chemotherapy and immune checkpoint blockade.

However, systemic delivery of protein therapeutics such as GOx remains a major challenge due to rapid degradation, short circulation half-life, and immunogenicity, which result in poor intratumoral accumulation and potential off-target toxicity. To address these limitations, a variety of nanocarriers—including polymeric nanogels, liposomes, metal–organic frameworks (MOFs), and inorganic nanoparticles—have been investigated for GOx delivery.^20–29^ While these platforms can improve enzyme stability in circulation, they are often hindered by compromised formulation integrity, suboptimal biocompatibility, limited biodegradability, and insufficient tumor selectivity. To overcome these challenges, we developed a biodegradable, biocompatible, and pH-responsive lipid–gelatin–protamine (LGP) nanogel specifically engineered for tumor-selective protein delivery. This core–shell nanostructure features a gelatin-protamine hydrogel core—formed via electrostatic complexation at a water–oil interface—that effectively encapsulates and stabilizes GOx or other protein therapeutics. Surrounding this core is a PLGA-lipid shell that prolongs systemic circulation and promotes tumor accumulation via the enhanced permeability and retention (EPR) effect. In addition, hydrophobic small-molecule drugs such as doxorubicin (DOX) can be integrated into the lipid-PLGA matrix, enabling a combination therapy within a single nanoplatform. Exposure to the acidic tumor microenvironment induces protonation of acidic residues in gelatin B, disrupting the nanogel structure and triggering a selective release of GOx at the tumor site. This nanozyme markedly enhances GOx pharmacokinetics, tumor selectivity, and enzymatic stability in vivo.

The versatility of the LGP nanogel platform also allows for the delivery of a broad range of therapeutic agents—including chemotherapeutic DOX, the apoptosis-inducing ligand TRAIL, and immune checkpoint inhibitors such as anti-PD-1 antibodies—making it a clinically translatable platform to enhance treatment efficacy in HCC. In this study, we report a multifaceted nanomedicine strategy using nanozymes to overcome therapeutic resistance in HCC. As illustrated in Figure 1, GOx-loaded nanozymes (GOx-LGP) deplete intratumoral glucose, leading to metabolic starvation and immunometabolic modulation that sensitizes tumors to apoptosis and immune attack. This simultaneously generates reactive oxygen species (ROS), triggers endoplasmic reticulum (ER) stress, and activates both death receptor- and mitochondria-mediated apoptotic pathways, thereby sensitizing HCC cells to TRAIL and DOX. Concurrently, TRAIL-loaded LGP nanogels (TRAIL-LGP) activate death receptors DR4/DR5 on HCC cells, amplifying extrinsic apoptotic signaling. The combination of GOx-LGP and TRAIL-LGP significantly induces apoptosis, suppresses primary tumor growth, and inhibits distal lung metastasis in orthotopic HCC models, resulting in markedly improved survival outcomes. Furthermore, DOX, an inducer of DNA damage and mitochondrial apoptosis, also promotes immunogenic cell death (ICD).^30^ When coformulated with GOx in GOx-DOX-LGP, the nanogel induces both apoptosis and ICD, amplifies antitumor immune responses, and enhances tumor sensitivity to immune checkpoint blockade. Finally, the combination of GOx-DOX-LGP and anti-PD-1 antibodies delivered via LGP nanogels (aPD1-LGP) synergistically reprograms the immunosuppressive tumor microenvironment, resulting in potent and sustained antitumor immunity. Collectively, these findings demonstrate the potential of GOx-based nanomedicine cocktails in overcoming resistance mechanisms and advancing treatment strategies for HCC.

**Figure 1:**
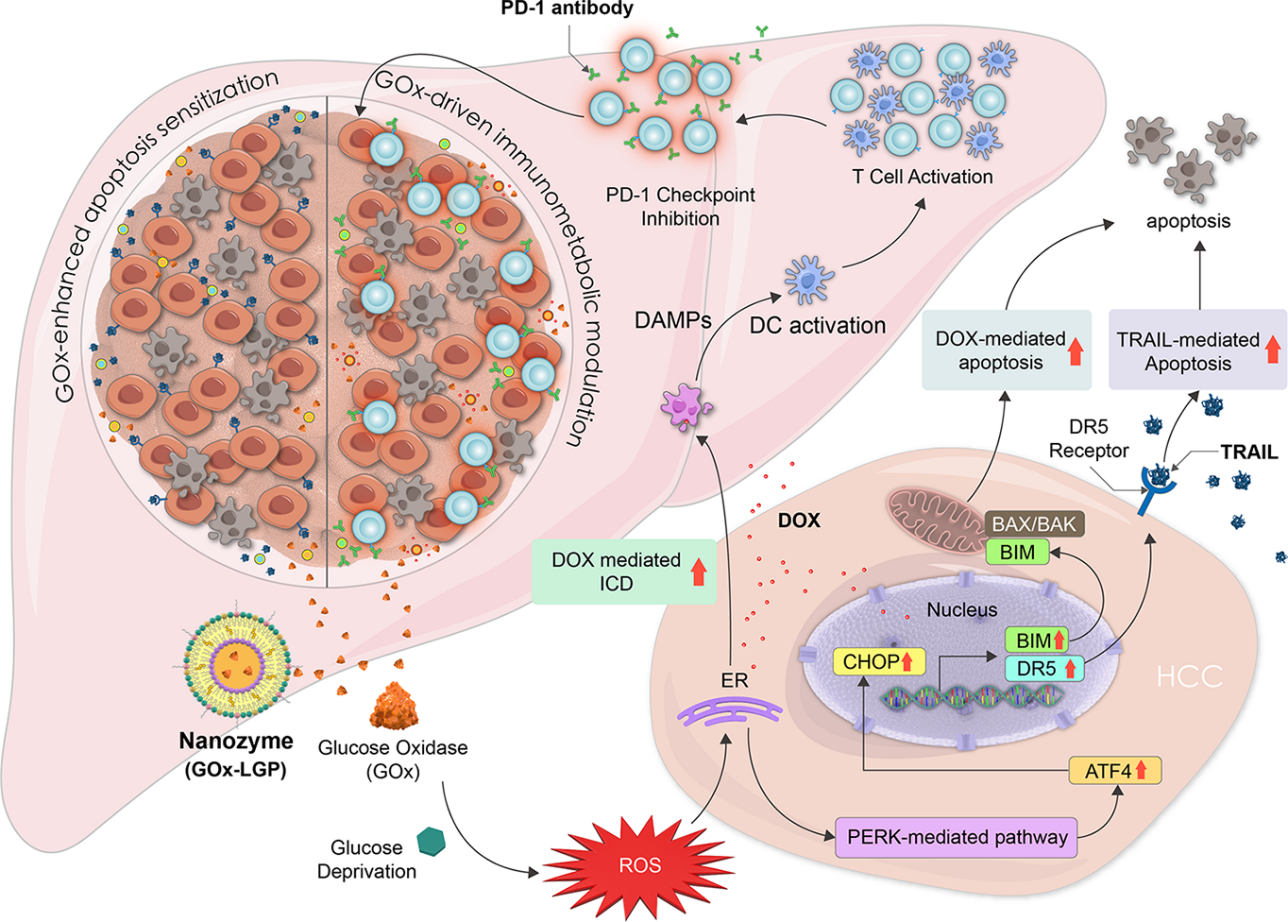
Schematic illustration of the mechanism by which GOx-loaded LGP nanogels promote apoptosis and anticancer immunity in HCC. Glucose oxidase (GOx)-loaded lipid–gelatin–protamine nanogels (GOx-LGP) disrupt the glycolytic metabolism of HCC by depleting intratumoral glucose, leading to metabolic starvation. The oxidation of glucose by GOx concurrently generates hydrogen peroxide (H_2_O_2_), elevating reactive oxygen species (ROS) and inducing endoplasmic reticulum (ER) stress. These metabolic perturbations sensitize HCC cells to apoptosis by upregulating death receptor 5 (DR5) and lowering the apoptotic threshold. When codelivered with the apoptosis-inducing ligand TRAIL via TRAIL-LGP nanogels, this strategy activates the extrinsic (death receptor-mediated) apoptotic pathway, while combination with doxorubicin (DOX) further engages intrinsic (mitochondrial) apoptosis. Co-delivery of GOx and DOX further induced immunometabolic modulation, which amplified immunogenic cell death, enhancing DAMP release, dendritic cell activation, and CD8^+^ T-cell-mediated antitumor immunity. The addition of immune checkpoint blockade (anti-PD-1 antibody) amplifies this immune response, establishing potent antitumor immunity. The LGP nanogel delivery platform enables tumor-selective and pH-responsive release of GOx, TRAIL, DOX, and anti-PD-1, providing a modular strategy that integrates apoptotic sensitization and immunometabolic modulation for the treatment of advanced HCC.

## 2 Results

### 2.1 Glycolysis Is a Dominant Metabolic Signature Associated with HCC Progression and Poor Prognosis

Gene set enrichment analysis of transcriptomic profiles from the TCGA-LIHC cohort revealed that HCC tumors exhibit robust activation of multiple oncogenic pathways, with glycolysis ranking among the most significantly upregulated metabolic signatures (Figure 2A). The Hallmark Glycolysis gene set displayed a marked enrichment in tumor tissues relative to adjacent normal liver (enrichment score ≈2.4; FDR *q* < 0.01), indicative of a metabolic shift toward increased glucose utilization. Notably, glycolytic pathway activity was elevated even in early-stage HCC and increased progressively with the tumor stage (Figure 2B), implicating glycolytic reprogramming as an early and escalating feature of HCC pathogenesis. Clinically, elevated glycolysis was associated with reduced patient survival. Kaplan–Meier analysis stratified by glycolysis gene set scores showed that patients with glycolysis-high tumors had significantly shorter disease-specific survival compared to those with lower scores (HR = 1.67, 95% CI 1.07–2.60; *P* = 0.028) (Figure 2C). These findings support the notion that enhanced glycolytic activity contributes to a more aggressive tumor phenotype.

**Figure 2:**
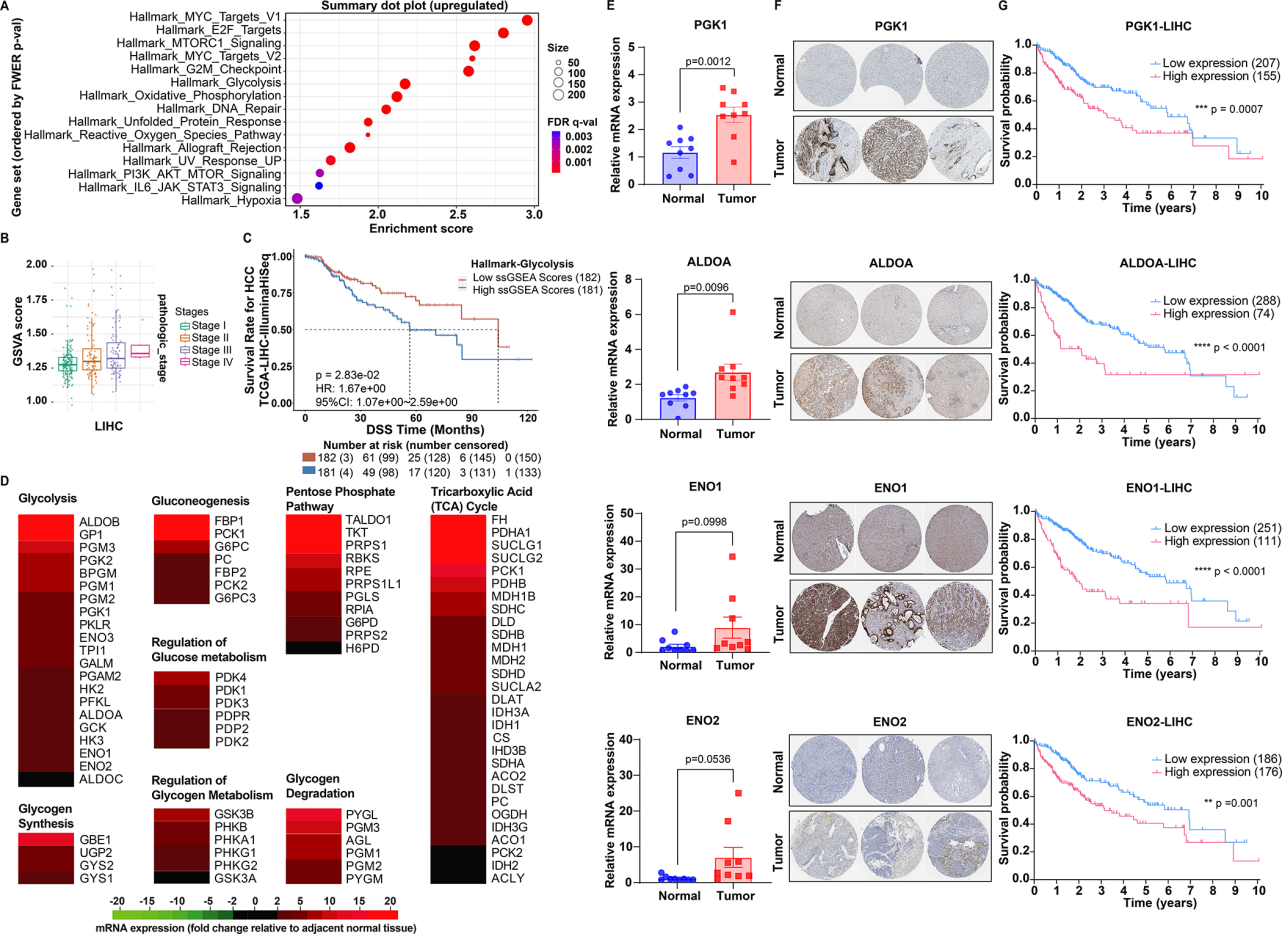
High expression of glycolysis-related genes in HCC patient samples. (A) Gene set enrichment analysis of transcriptomic profiles from the TCGA-LIHC reveals glycolysis ranking among the most significantly upregulated metabolic signatures. (B) Gene Set Variation Analysis (GSVA) scores across stages of LIHC reveal elevated pathway activity in higher pathologic stages, indicating a positive correlation between the gene set and LIHC progression. (C) Kaplan–Meier analysis of disease-specific survival (DSS) in TCGA-LIHC patients stratified by ssGSEA scores for the glycolysis gene set. (D) mRNA expression levels of 84 key genes involved in glucose metabolism were analyzed in HCC patient samples using the RT^2^ Profiler PCR Array. Data are presented as fold changes relative to the corresponding levels in adjacent normal tissue samples (*n* = 3). (E) Upregulated mRNA expression level of glycolysis-related genes including *PGK1, ALDOA, ENO1,* and *ENO2* in HCC patient tumor samples compared to adjacent normal tissue samples (*n* = 9). (F) Immunohistochemistry (IHC) images retrieved from the Human Protein Atlas (HPA) database revealed higher protein expression levels of PGK1, ALDOA, ENO1, and ENO2 in HCC tissues compared to normal liver. (G) Overall survival analysis of TCGA-LIHC patients using GSCA showed that high expression of PGK1, ALDOA, ENO1, and ENO2 was individually associated with poorer prognosis. Data in panels (A–C) were analyzed using the GSCA platform; protein expression data in panels F and survival data in panel G were accessed via the Human Protein Atlas. Data are the mean values ± S.E.M.

To validate this transcriptional reprogramming in clinical specimens, we performed a targeted PCR array analysis on paired HCC tumors and adjacent normal tissues from three patients. The results demonstrated a coordinated upregulation of genes involved in diverse branches of glucose metabolism—including glycolysis, gluconeogenesis, the pentose phosphate pathway, glycogen metabolism, and the tricarboxylic acid (TCA) cycle (Figure 2D and Table S1). These findings suggest global metabolic rewiring in HCC, with activation extending beyond glycolysis to encompass broader glucose utilization pathways. Notably, glycolytic enzymes such as *PGK1*, *ALDOA*, *ENO1*, and *ENO2* were markedly elevated, reflecting enhanced glycolytic flux. These findings were confirmed in an independent cohort using qRT-PCR, which showed significantly higher expression of *PGK1*, *ALDOA*, and *ENO2* in tumors compared to normal liver (Figure 2E). Immunohistochemistry data from the Human Protein Atlas further confirmed increased protein levels of these enzymes in HCC samples (Figure 2F).

Finally, analysis of TCGA patient survival data revealed that the high expression of *PGK1*, *ALDOA*, *ENO1*, and *ENO2* individually predicted poorer prognosis, with significant reductions in overall survival in high-expression groups across all four genes (Figure 2G). These findings identify glycolytic reprogramming as a hallmark of HCC progression and a potential therapeutic vulnerability that may be exploited in future therapeutic strategies for HCC.

### 2.2 GOx-Induced Metabolic Stress Enhances Apoptotic Sensitivity in HCC Cells

Based on the aforementioned findings, we hypothesized that disrupting glycolysis and glucose metabolism could induce metabolic stress and sensitize HCC cells to therapeutic agents. To test this, we enzymatically depleted glucose using GOx, which not only consumes glucose but also produces ROS (H_2_O_2_), thereby simultaneously starving cells and generating oxidative stress. GOx treatment induced a dose-dependent increase in intracellular ROS levels in both murine (HCA-1) and human (Hep3B) HCC cells, as determined by dichlorofluorescein (DCF) fluorescence (Figures 3A and S1). This oxidative stress triggered pro-apoptotic signaling pathways. Notably, GOx upregulated the endoplasmic reticulum (ER) stress-associated ATF4/CHOP axis and death receptor DR5 in a dose-dependent manner (Figure 3B). In parallel, GOx suppressed Bim phosphorylation, resulting in elevated Bim levels and activation of the intrinsic apoptosis pathway (Figure 3B), indicating that both extrinsic and intrinsic apoptotic programs are engaged following glucose deprivation (Figure 3C).

**Figure 3:**
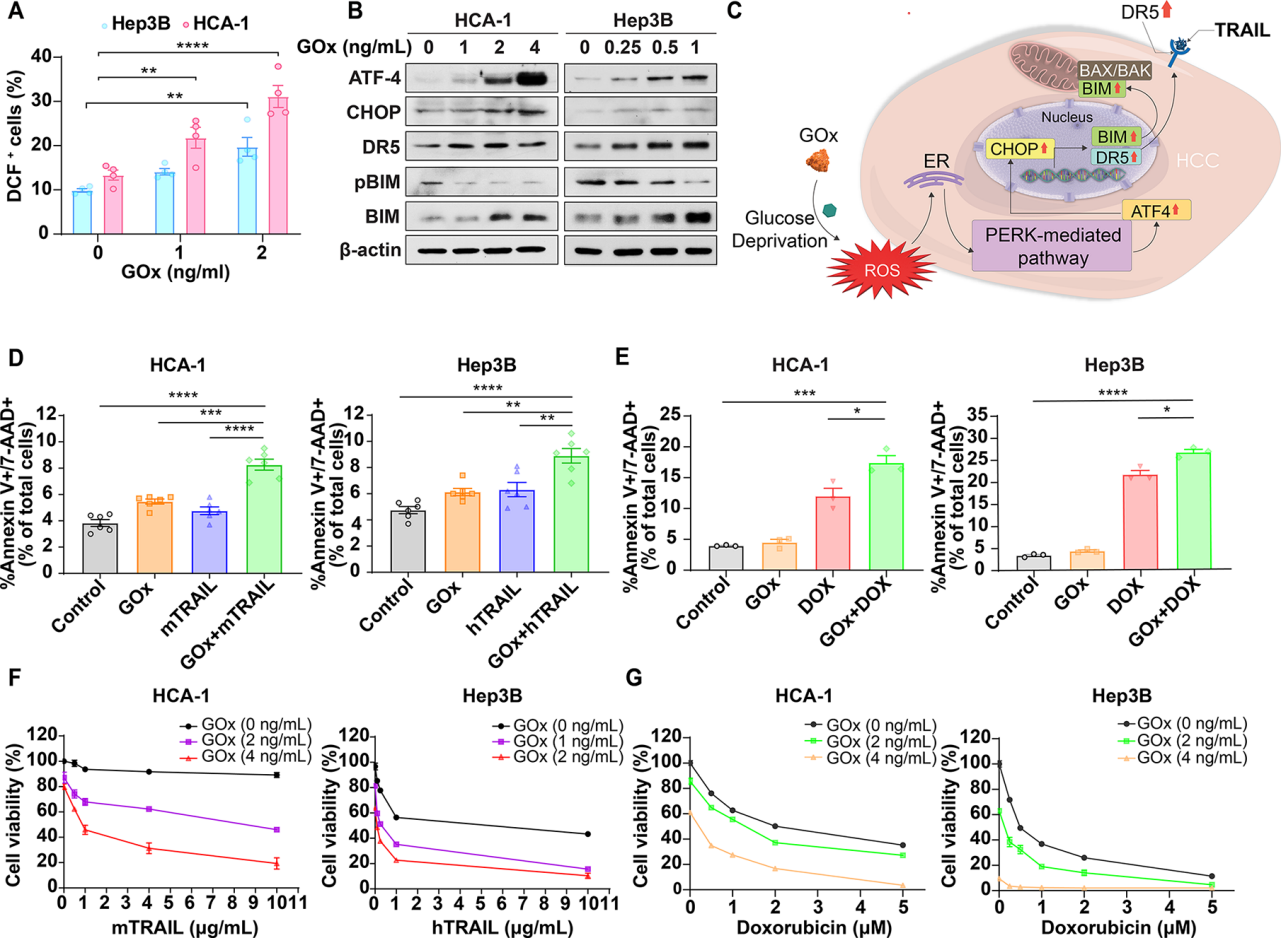
GOx induces metabolic stress and enhances apoptosis sensitivity in HCC cells. (A) Intracellular reactive oxygen species (ROS) levels in murine (HCA-1) and human (Hep3B) HCC cells following a 3 h treatment with GOx, measured by H_2_DCFDA fluorescence and flow cytometry (*n* = 4). (B) Western blot analysis of apoptotic signaling pathways in HCA-1 and Hep3B cells after 24 h GOx exposure, showing dose-dependent activation of ER stress markers (ATF4, CHOP), upregulation of death receptor 5 (DR5), suppression of Bim phosphorylation, and accumulation of total Bim protein. (C) Schematic overview of GOx-mediated induction of both extrinsic and intrinsic apoptotic pathways under glucose deprivation. (D, E) Flow cytometry analysis of apoptosis following 24 h treatment with GOx in combination with TRAIL (D) or doxorubicin (DOX) (E) in HCA-1 and Hep3B cells, assessed by Annexin V-FITC and 7-AAD staining (*n* = 3–6). (F, G) Cell viability analysis of HCA-1 and Hep3B cells treated with increasing concentrations of GOx in combination with either TRAIL (F) or DOX (G), measured by MTT assay (*n* = 6). Data are the mean values ± S.E.M., *p < 0.05, **p < 0.01, ***p < 0.001, ****p < 0.0001.

To determine whether GOx-induced stress sensitizes HCC cells to therapeutic agents, we evaluated their response to the death ligand TRAIL and the chemotherapeutic doxorubicin (DOX). In both HCA-1 and Hep3B cells, cotreatment with GOx significantly enhanced TRAIL-induced apoptosis, as evidenced by an increased fraction of Annexin V^+^/7-AAD^+^ cells compared to either agent alone (Figure 3D). Similarly, GOx markedly potentiated DOX-induced apoptosis, with the combination treatment yielding a higher apoptotic fraction than monotherapies (Figure 3E). Cell viability assays further confirmed these findings. In both murine and human HCC cells, GOx dose-dependently reduced viability in the presence of TRAIL (Figure 3F) and DOX (Figure 3G), indicating synergistic cytotoxicity (Figure S2). Together, these results support a mechanism in which glucose deprivation and concomitant ROS generation activate pro-apoptotic signaling pathways, thereby sensitizing HCC cells to TRAIL and DOX. This combinatorial strategy offers a promising metabolic vulnerability to enhance therapeutic efficacy against HCC.

### 2.3 pH-Responsive Nanogel System Enhances Tumor Selectivity and Stability of Protein Therapeutics

Despite their therapeutic promise, the clinical utility of GOx and other macromolecular cancer therapeutics has been hindered by rapid systemic clearance, poor tumor accumulation, and dose-limiting toxicities. To address these challenges and enable tumor-selective, stimuli-responsive delivery, we developed an LGP nanogel system for the encapsulation and controlled release of protein therapeutics such as GOx and TRAIL (Figure 4A). In this formulation, proteins are first encapsulated within a gelatin B-protamine hydrogel core formed via a water-in-oil emulsion. The cationic headgroups of DOTAP lipids interact electrostatically with the negatively charged gelatin at the interface, while the hydrophobic lipid tails facilitate solubilization in nonpolar solvents, such as cyclohexane. These emulsion-mediated interfacial and confinement effects promote spontaneous gelation of the biopolymer complex without chemical cross-linkers. The resulting protein-loaded cores are then wrapped in a lipid-PLGA shell, forming stable, pH-sensitive nanogels. SEM and TEM analyses confirmed the spherical morphology of GOx-LGP nanogels, with an average diameter of 172.7 ± 1.7 nm, a PDI of 0.167 ± 0.015, a near-neutral zeta potential (−0.95 ± 0.36 mV), and a protein encapsulation efficiency of 64.2 ± 4.4% (Figures 4B, S3 and Table S2). In vitro release studies demonstrated minimal protein release at physiological pH 7.4 but significantly accelerated release under mildly acidic conditions (pH 5.5–6.5), consistent with gelatin B protonation–induced disassembly of the gelatin–protamine matrix (Figure 4C). This pH-responsive behavior enables selective cargo release in the acidic tumor microenvironment. Enzymatic activity assays revealed that GOx-LGP retained a catalytic function comparable to free GOx over extended time courses, with only a modest initial lag, indicating that the nanogel formulation preserves functional protein activity (Figure 4D).

**Figure 4:**
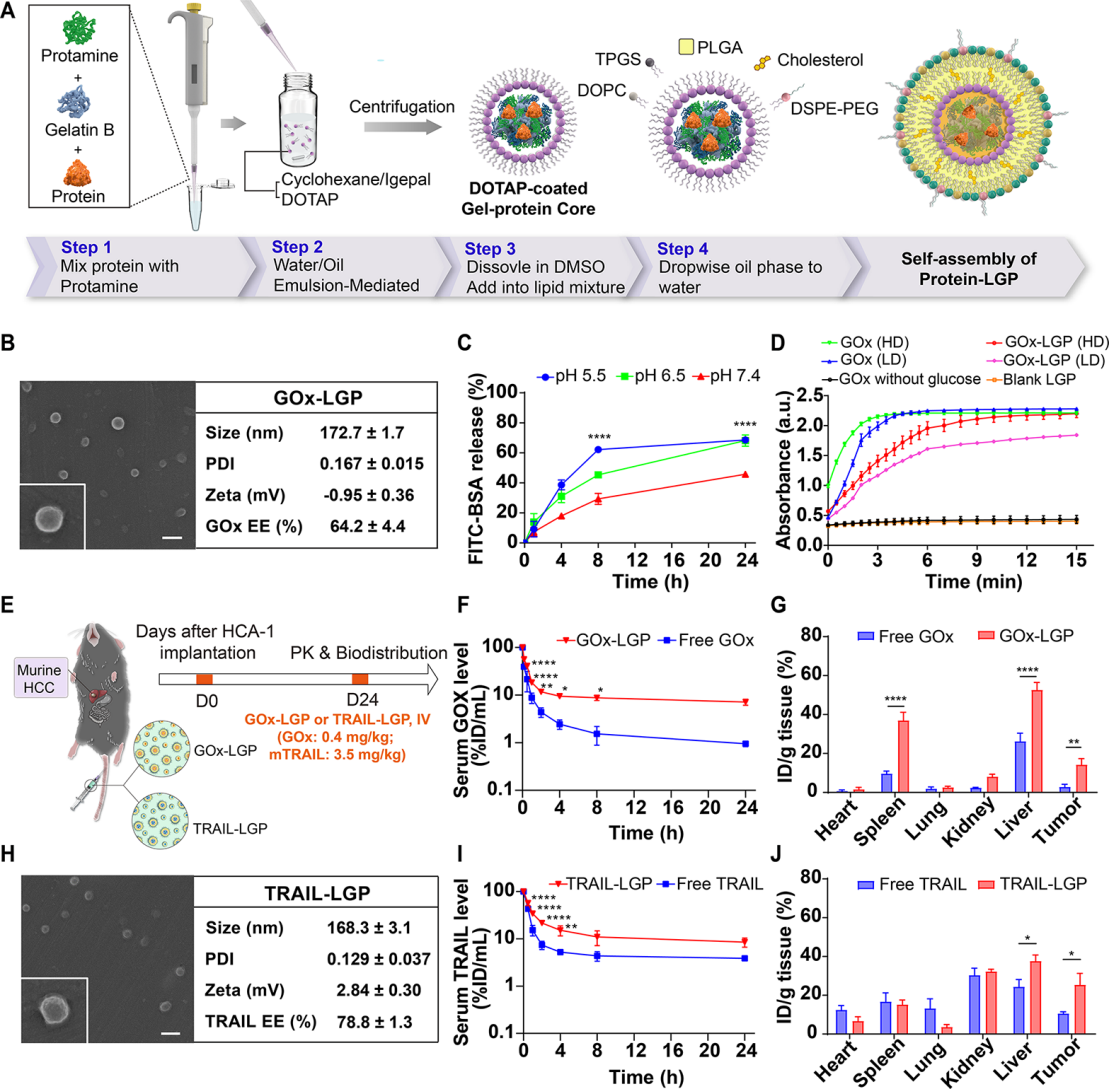
Physicochemical characterization, enzymatic activity, and biodistribution of GOx-LGP and TRAIL-LGP nanogels. (A) Schematic overview of the LGP nanogel formulation. Therapeutic proteins were first encapsulated within a gelatin–protamine hydrogel core formed via water-in-oil emulsion and electrostatic complexation. The core was then coated with DOTAP lipid and subsequently integrated into a PLGA–lipid shell to form stable, pH-responsive LGP nanogels. (B) Representative SEM image (scale bar: 200 nm), hydrodynamic diameter, polydispersity index (PDI), zeta potential, and encapsulation efficiency of GOx-LGP nanogels (*n* = 3). (C) *In vitro* release profile of FITC-BSA (used as a model protein) from LGP nanogels in buffers of varying pH (5.5–7.4), demonstrating accelerated release under mildly acidic conditions (*n* = 3). (D) Retained enzymatic activity of GOx-LGP compared to free GOx over time, assessed by H_2_O_2_ generation (*n* = 3). GOx activity was quantified by measuring absorbance at 595 nm following treatment with high dose (HD, 1.5 μg/mL) and low dose (LD, 0.5 μg/mL) of GOx. (E) Experimental protocol for pharmacokinetic and biodistribution studies. At 24 days postimplantation, mice bearing orthotopic HCA-1 tumors were intravenously injected with Alexa488-labeled GOx or TRAIL, in free or LGP-encapsulated form (protein dose: GOx, 0.4 mg/kg; TRAIL, 3.5 mg/kg). Fluorescence intensity in homogenized tissue lysates was measured 4 h post-injection. (F) Pharmacokinetic profiles of FITC-labeled GOx in free form and in GOx-LGP nanogels following intravenous injection (*n* = 3). (G) Biodistribution of GOx-LGP versus free GOx across tissues, presented as percentage of injected dose per gram (ID/g) (*n* = 4). (H) Representative SEM image (scale bar: 200 nm), hydrodynamic diameter, PDI, zeta potential, and encapsulation efficiency of TRAIL-LGP nanogels (*n* = 3). (I) Pharmacokinetic profiles of FITC-labeled TRAIL in free form and in TRAIL-LGP nanogels following intravenous injection (*n* = 3). (J) Biodistribution of TRAIL-LGP versus free TRAIL across tissues (ID/g) 4 h postinjection (*n* = 4). Data in panels A and H are the mean values ± S.D.; data in other panels are the mean values ± S.E.M., *p < 0.05, **p < 0.01, ****p < 0.0001.

We next evaluated the pharmacokinetic and biodistribution profiles of GOx and TRAIL delivered via LGP nanogels in vivo (Figure 4E). Following intravenous (IV) administration in orthotopic murine HCC models (HCA-1), Alexa-488-labeled GOx-LGP exhibited significantly prolonged circulation time relative to free GOx (Figure 4F), accompanied by enhanced accumulation in tumors, liver, and spleen at 4 h postinjection (Figure 4G). These findings suggest that LGP nanogels not only extend systemic half-life but also enhance tumor delivery, likely via the enhanced EPR effect. Similarly, TRAIL-loaded LGP nanogels (TRAIL-LGP) displayed spherical morphology by SEM and TEM, with an average particle size of 168.3 ± 3.1 nm, a PDI of 0.129 ± 0.037, a zeta potential of 2.84 ± 0.30 mV, and high encapsulation efficiency (78.8 ± 1.3%) (Figures 4H, S3 and Table S2). TRAIL-LGP demonstrated improved pharmacokinetic properties compared to free TRAIL (Figure 4I), and there was a marked increase in intratumoral TRAIL accumulation (Figure 4J). Together, these data highlight the utility of the LGP nanogel platform for enhancing the bioavailability and tumor targeting of protein therapeutics, supporting its potential for improving therapeutic outcomes in HCC.

### 2.4 Enhanced Apoptosis and Tumor Suppression by GOx- and TRAIL-Loaded Nanogels in Orthotopic Murine and Human HCC Models

Having demonstrated improved tumor targeting delivery of LGP nanogels, we next evaluated the therapeutic efficacy of nanoscale GOx and TRAIL in HCC. *In vitro*, the co-administration of GOx-LGP and TRAIL-LGP induced robust apoptosis in both murine (HCA-1) and human (Hep3B) HCC cells, as evidenced by significantly increased Annexin V/PI staining compared to either monotherapy (Figure 5A). Consistent with the effects of free-form GOx shown in Figure 3, GOx-LGP treatment elicited pronounced cellular stress responses, characterized by elevated expression of ER stress markers, including ATF4 and CHOP. This stress response led to the upregulation of DR5—a critical mediator of TRAIL-induced apoptosis—in both HCA-1 and Hep3B cells (Figure 5B).

**Figure 5:**
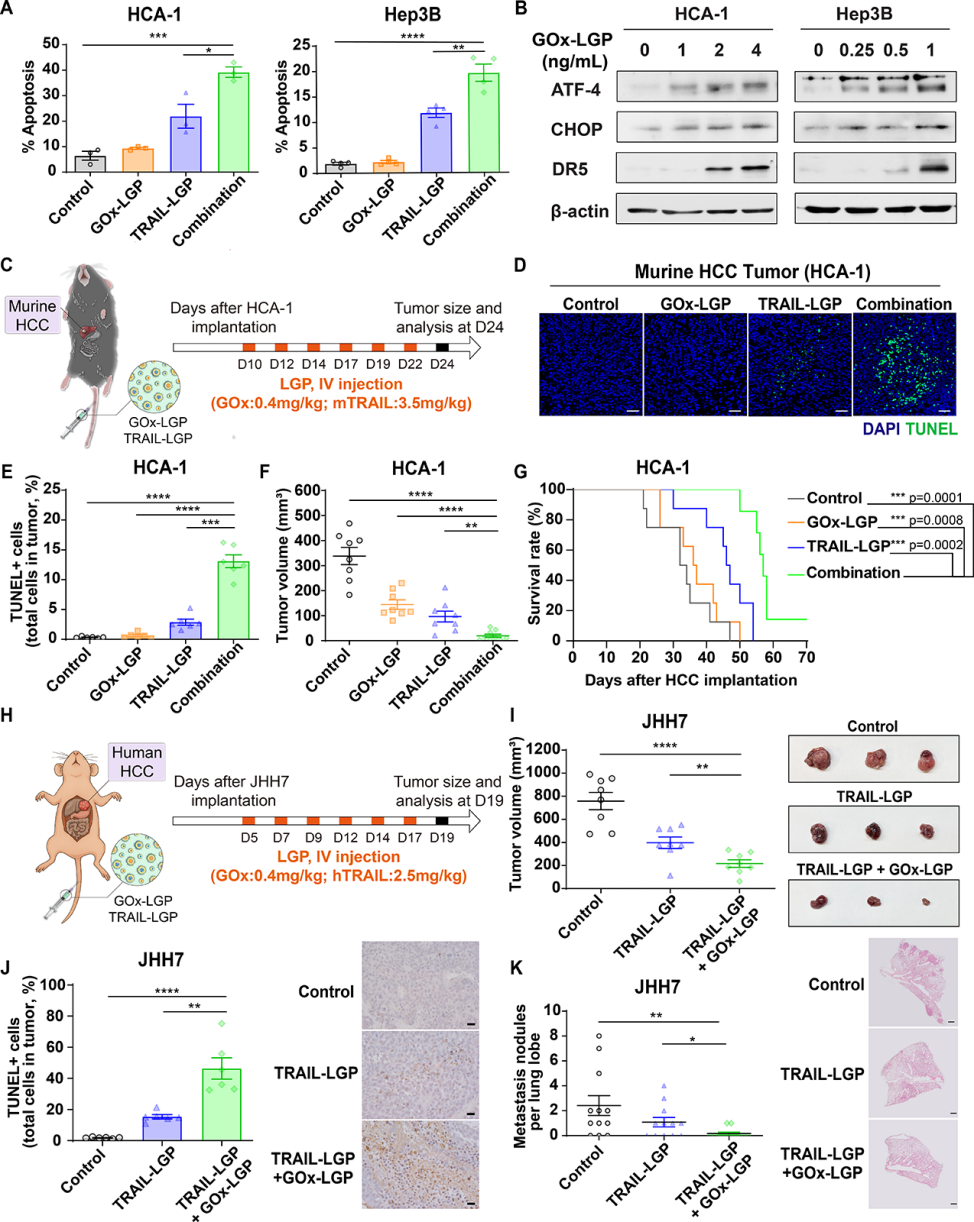
Synergistic induction of apoptosis and tumor suppression by GOx-LGP and TRAIL-LGP nanogels in murine and human HCC models. (A) Flow cytometry analysis of apoptosis in murine (HCA-1) and human (Hep3B) HCC cells following 24-h treatment with GOx-LGP, TRAIL-LGP, or their combination, as assessed by Annexin V-FITC staining (*n* = 3–4). (B) Western blot analysis showing upregulation of DR5 and other key death receptor pathway components in HCC cells after 24-h treatment with GOx-LGP, highlighting glucose deprivation-induced sensitization to TRAIL-mediated apoptosis. (C) Schematic representation of the treatment protocol in the syngeneic murine HCA-1 orthotopic HCC model. Ten days post tumor implantation, mice received six intravenous doses of either GOx-LGP (0.4 mg/kg), TRAIL-LGP (3.5 mg/kg), or both in combination at 2–3 day intervals; tumors were analyzed on day 24. (D) Representative immunofluorescence images of TUNEL staining in tumor sections, indicating apoptotic cell death (scale bar: 50 μm). (E) Quantification of TUNEL^+^ cells in HCA-1 tumors harvested 24 days post-implantation (*n* = 6). (F) Tumor volume changes in response to different treatments (*n* = 8). (G) Kaplan–Meier survival analysis of mice bearing orthotopic HCA-1 tumors treated with monotherapy or combination therapy (*n* = 8). (H) Schematic illustration of the treatment regimen for the orthotopic human JHH7 xenograft model. Mice were treated intravenously six times with GOx-LGP (0.4 mg/kg), TRAIL-LGP (2.5 mg/kg), or both, starting 5 days post tumor implantation and analyzed on day 19. (I) Representative tumor images and quantification of tumor volumes in JHH7-bearing mice (*n* = 8). (J) Quantification of TUNEL^+^ cells in JHH7 tumors (*n* = 6) and representative colorimetric images of TUNEL-stained sections (scale bar: 20 μm). (K) Quantification and representative H&E images of lung metastatic nodules in JHH7-bearing mice, showing reduced metastatic burden after combination treatment (scale bar: 500 μm; *n* = 4). Data are the mean values ± S.E.M., *p < 0.05, **p < 0.01, ***p < 0.001, ****p < 0.0001.

We next assessed therapeutic efficacy *in vivo* by using orthotopic HCC models. In a syngeneic murine model established through intrahepatic implantation of HCA-1 cells (Figure 5C), IV administration of the combined GOx-LGP and TRAIL-LGP treatment significantly enhanced tumor apoptosis, as evidenced by TUNEL staining (Figure 5D,E). This combination therapy also resulted in significant tumor growth inhibition (Figures 5F and S4) and prolonged survival (Figure 5G) compared to those of monotherapies (GOx-LGP or TRAIL-LGP alone) or control groups, highlighting the potent synergy between GOx-induced metabolic stress and TRAIL-mediated apoptosis.

To assess translational relevance, we further examined therapeutic responses in an orthotopic xenograft model bearing human JHH-7 HCC tumors (Figure 5H). The combination of GOx-LGP and TRAIL-LGP significantly suppressed tumor growth (Figures 5I and S5) and enhanced intratumoral apoptosis (Figure 5J), compared with either agent alone. Notably, the combination treatment also led to near-complete inhibition of lung metastases (Figure 5K), as quantified by metastatic burden analysis. Collectively, these findings show the synergistic antitumor efficacy of LGP-mediated delivery of GOx and TRAIL—potentiating apoptosis through metabolic and death receptor pathways, suppressing both primary and metastatic tumor progression, and prolonging survival in both murine and human HCC models.

### 2.5 GOx Enhances DOX-Induced Apoptosis and Immunogenic Cell Death to Potentiate Antitumor Immunity in HCC

Building upon these findings, we next examined whether GOx could similarly enhance the efficacy of DOX, a DNA-damaging chemotherapeutic that induces intrinsic apoptotic signaling. To achieve this, we developed a dual-compartment nanozyme (GOx-DOX-LGP) encapsulating GOx in a gelatin-protamine hydrogel core and DOX in the outer PLGA shell (Figure 6A). The resulting nanogels exhibited a uniform spherical morphology (181.6 ± 2.5 nm), low polydispersity (PDI = 0.20 ± 0.01), and a near-neutral surface charge (−3.67 ± 0.10 mV), with encapsulation efficiencies of 43.8 ± 1.7% for DOX (Figures 6A, right and S3). Both cargos demonstrated pH-responsive release, with accelerated DOX release under mildly acidic conditions (pH 5.5–6.5) compared to physiological pH, indicating tumor-selective release behavior (Figure 6B).

**Figure 6:**
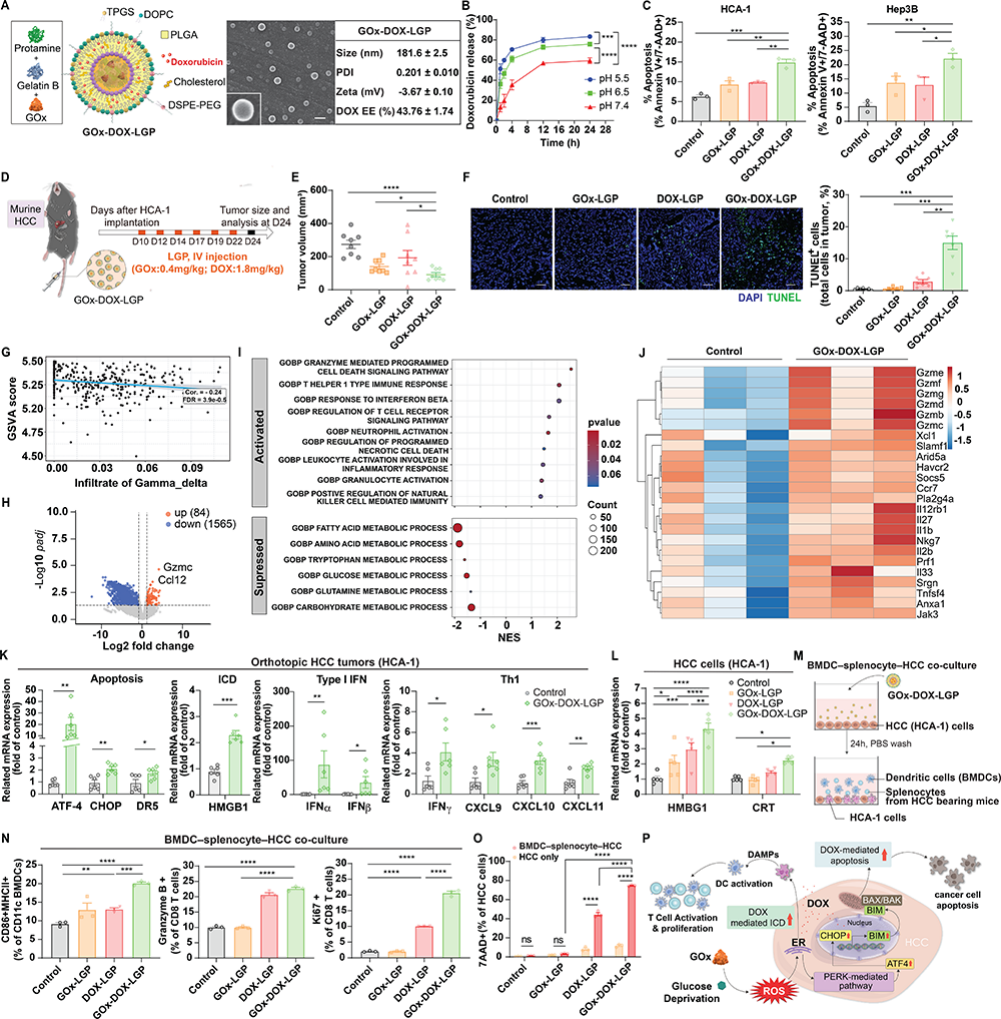
LGP-mediated co-delivery of GOx and DOX synergistically enhances apoptosis and immunogenic cell death, amplifying antitumor immunity in HCC. (A) Schematic illustration of GOx-DOX-LGP structure, showing dual-compartment encapsulation of GOx (core) and DOX (shell). SEM image (scale bar: 200 nm), hydrodynamic size, polydispersity index (PDI), zeta potential, and encapsulation efficiency (EE, %) of GOx-DOX-LGP are shown. (B) pH-responsive DOX release profile from GOx-DOX-LGP in buffers ranging from pH 5.5 to 7.4 (*n* = 3). (C) Apoptosis induction in HCA-1 and Hep3B cells after 24 h treatment with GOx-LGP, DOX-LGP, or GOx-DOX-LGP, assessed by Annexin V-FITC/7-AAD flow cytometry (*n* = 3). (D) Experimental protocol: 10 days after orthotopic implantation of HCA-1 tumors, mice were intravenously treated with GOx-DOX-LGP (GOx: 0.4 mg/kg; DOX: 1.6 mg/kg), GOx-LGP (0.4 mg/kg), or DOX-LGP (1.8 mg/kg) six times at 2–3 day intervals. Tumors were harvested on day 24. (E) Tumor volume measurements of orthotopic HCC tumors following treatment (*n* = 8). (F) Representative TUNEL-stained tumor sections (scale bar: 50 μm) and quantification of TUNEL^+^ apoptotic cells in HCA-1 tumors (*n* = 7). (G) Spearman correlation analysis in the TCGA–LIHC data set showing inverse correlation between glycolysis gene set variation analysis (GSVA) scores and γδ T cell infiltration, indicating a glycolysis-driven immunosuppressive microenvironment. (H) Transcriptomic profiling of HCC tumors treated with GOx-DOX-LGP *in vivo* (*n* = 3). Volcano plot of differentially expressed genes (DEGs) relative to untreated controls. (I) Gene ontology analysis highlighting immune activation and metabolic suppression. (J) Heatmap of immune-related gene expression in treated versus control tumors. (K) RT-qPCR analysis of pro-apoptotic (ATF4, CHOP, DR5), ICD-associated (HMGB1), and immune-related genes (type I interferons and Th1 cytokines) in HCC tumors treated with GOx-DOX-LGP, compared to untreated controls (*n* = 6). (L) RT-qPCR quantification of ICD-associated DAMPs (e.g., HMGB1, calreticulin) in HCA-1 cells in vitro after 24-h treatment with GOx-LGP, DOX-LGP, or GOx-DOX-LGP (*n* = 5). (M) Diagram of *in vitro* co-culture model comprising DCs, splenocytes from HCA-1 tumor-bearing mice, and HCA-1 tumor cells to assess GOx-DOX-LGP-induced ICD and immune activation. (N) Flow cytometry analysis of DC maturation markers (MHC-II, CD86) in CD11c^+^ BMDCs, and granzyme B and Ki67 expression in CD8^+^ T cells, following 24-h treatment in the coculture system (*n* = 3). (O) Tumor cell lysis in co-culture conditions was assessed by 7-AAD staining via flow cytometry (*n* = 3). (P) Schematic illustration of the proposed mechanism: GOx-induced metabolic and ER stress enhances DAMP release and ICD, leading to DC activation, T-cell priming, and robust adaptive immune responses. Data in panels A are the mean values ± S.D.; data in other panels are the mean values ± S.E.M., *p < 0.05, **p < 0.01, ***p < 0.001, ****p < 0.0001.

*In vitro,* GOx-DOX-LGP induced significantly greater apoptosis than did monotherapies in HCA-1 and Hep3B cells (Figure 6C). In orthotopic HCA-1 tumor models, systemic administration of GOx-DOX-LGP led to pronounced tumor growth inhibition (Figure 6D,E) and enhanced apoptosis, as evidenced by TUNEL-positive staining in tumor sections (Figure 6F), further confirming its therapeutic superiority.

Enhanced glycolysis in HCC not only fuels tumor growth but also drives immunometabolic suppression by creating a lactate-rich tumor microenvironment and impairing cytotoxic T-cell function.^8–10^ To investigate this immunometabolic barrier, we examined the correlation between glycolytic activity and immune infiltration in patient-derived HCC data sets from the TCGA–LIHC cohort. Notably, the glycolysis-related genes ALDOA, PGK1, and ENO2—previously shown to be upregulated in HCC tumors (Figure 2)—were inversely correlated with γδ T cell infiltration (Spearman’s *r* = −0.24, FDR = 3.9e–05; Figure 6G), implicating glycolytic reprogramming as a barrier to effective immune infiltration. These findings suggest that metabolic modulation via GOx may reprogram tumor immunometabolism and enhance antitumor immune responses. To further explore this hypothesis, we assessed transcriptomic changes following GOx-DOX-LGP treatment in murine HCC tumors. Transcriptomic profiling of GOx-DOX-LGP-treated tumors revealed 1649 differentially expressed genes (DEGs), including 84 upregulated and 1565 downregulated transcripts (Figure 6H). Gene ontology enrichment analysis showed downregulation of glucose and fatty acid metabolic pathways alongside marked upregulation of immune-related pathways, including cytokine production, leukocyte activation, and antigen presentation (Figure 6I and Table S3). A heatmap of immune-related genes further demonstrated robust activation of cytotoxic immune programs, characterized by elevated expression of granzymes (Gzmb and Gzme), perforin (Prf1), and key mediators of T cell effector function (Il2b, Xcl1, and Slamf1), along with innate immune regulators such as Il1b, Il33, and Nkg7 (Figure 6J). Real-time PCR analysis of HCC tumors confirmed that GOx-DOX-LGP treatment upregulated pro-apoptotic genes (ATF4, CHOP, DR5), the ICD-associated molecule HMGB1, and proinflammatory mediators, including type I interferons and Th1-associated cytokines (Figure 6K), indicating enhanced immunostimulatory activity.

To further validate the underlying mechanisms *in vitro*, we assessed the ICD induction and immune activation. GOx-DOX-LGP treatment markedly upregulated DAMPs, including HMGB1 and calreticulin (CRT), compared to single-agent controls (Figure 6L). In a BMDC-splenocyte-HCC co-culture system (Figure 6M), GOx-DOX-LGP significantly enhanced dendritic cell maturation (CD86^+^ MHC-II^+^ in CD11c^+^) and CD8^+^ T-cell activation and proliferation, as evidenced by increased granzyme B and Ki67 expression (Figure 6N), culminating in enhanced tumor cell lysis (Figure 6O). A mechanistic schematic summarizes these effects: GOx-induced metabolic and ER stress amplifies DAMP release and ICD, facilitating DC activation and T-cell priming and collectively reshaping the tumor microenvironment to support adaptive antitumor immunity (Figure 6P).

### 2.6 Combination of Glucose Metabolism Reprogramming and Checkpoint Blockade via Nanogels Enhances Antitumor Responses in HCC

Given the potential immunometabolic modulation induced by GOx-DOX-LGP, we hypothesized that its combination with immune checkpoint blockade could further modulate the immunosuppressive tumor microenvironment and amplify T-cell-mediated antitumor responses. To test this, we formulated anti-PD-1 antibody-loaded nanogels (aPD1-LGP) by encapsulating the antibody within a gelatin-protamine hydrogel core and stabilizing the construct with a PLGA-based lipid shell (Figure 7A). The resulting nanogels exhibited a uniform spherical morphology, with an average diameter of 187.3 ± 3.2 nm, a PDI of 0.190 ± 0.017, and a zeta potential of −5.95 ± 0.32 mV. The encapsulation efficiency of anti-PD-1 antibody was 51.4 ± 4.9% (Figures 7A, right, S3 and Table S2).

**Figure 7:**
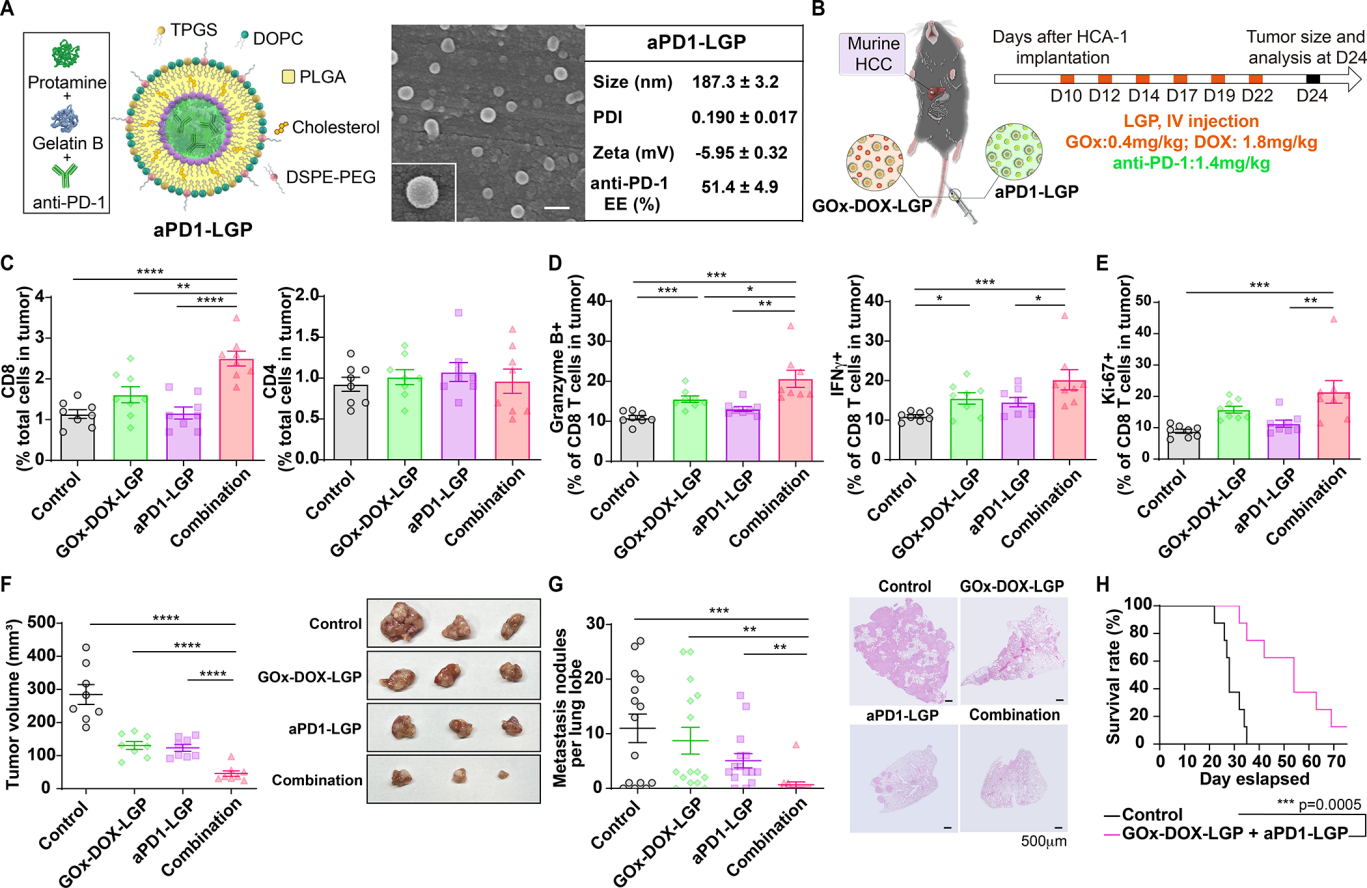
Combining GOx-DOX-LGP with PD-1 checkpoint inhibition effectively alters the tumor immune landscape, driving enhanced synergistic antitumor effects in HCC. (A) Illustration of nanogel structure, representative SEM image (scale bar = 200 nm), nanoparticles size, polydispersity index (PDI), zeta potential, and encapsulation efficiency (EE, %) of aPD1-LGP nanogels. (B) Schematic of the experimental protocol. Ten days after the implantation of HCA-1 cells, mice were intravenously treated with either GOx-DOX-LGP (GOx: ∼0.4 mg/kg; DOX: ∼1.8 mg/kg) or aPD-1-LGP (1.4 mg/kg) for single treatment groups, and both nanogels for combination treatment group six times (at 2–3*d* intervals), and tumors were harvested and analyzed on day 24. (C) Populations of cytotoxic CD8^+^ T lymphocytes (CD3+CD8+) and helper CD4^+^ T lymphocytes (CD3^+^CD4^+^) in tumors detected by flow cytometry (*n* = 8). (D) Percentages of activated granzyme B+ and IFN-γ+ cytotoxic CD8^+^ T lymphocytes detected by flow cytometry (*n* = 8). (E) Percentage of proliferating (Ki67+) cytotoxic CD8^+^ T lymphocytes detected by flow cytometry (*n* = 8). (F) Representative images and volumes of murine orthotopic HCC tumors in response to different treatments (*n* = 8). (G) Number of spontaneously occurring lung metastatic nodules in the orthotopic HCC model was reduced in mice treated with GOx-DOX-LGP and aPD1-LGP (*n* = 5), and representative H&E-stained images of metastatic tumor nodules in the lung are shown (Scale bar: 500 μm). (H) Combination of GOx-DOX-LGP and aPD1-LGP nanogels remarkably prolonged the overall survival in the orthotopic murine HCC model (*n* = 8). Data in panels A are the mean values ± S.D.; data in other panels are the mean values ± S.E.M., *p < 0.05, **p < 0.01, ***p < 0.001, ****p < 0.0001.

We next evaluated the therapeutic synergy of GOx-DOX-LGP and aPD1-LGP in an orthotopic, immunocompetent HCA-1 HCC model (Figure 7B). Mice receiving the combination therapy demonstrated a significant increase in intratumoral CD8^+^ T cell infiltration compared to the monotherapy or control groups (Figure 7C). Furthermore, the combination of GOx-DOX-LGP and aPD1-LGP further enhanced the activation of intratumoral CD8^+^ T cells, as indicated by increased granzyme B^+^ and IFN-γ^+^ populations (Figures 7D and S6), along with elevated proliferation marked by Ki67^+^ expression (Figures 7E and S6), suggesting improved effector T-cell-mediated antitumor immunity within the tumor microenvironment. In addition, GOx-DOX-LGP treatment increased the proportion of CD44^+^CD62L^+^ central memory CD8^+^ T cells in the spleen, indicative of durable systemic immune memory formation (Figure S7). This enhanced immune activation translated into substantial therapeutic benefits *in vivo*. The combination therapy significantly reduced primary tumor burden (Figures 7F, S8 and S9), suppressed lung metastasis (Figure 7G), and prolonged overall survival compared to the control (Figure 7H). Collectively, these results demonstrate that integrating GOx-DOX-LGP with PD-1 checkpoint inhibition effectively modulates the tumor immune landscape and promotes synergistic antitumor activity in HCC.

### 2.7 Evaluation of the Toxicity Profile of the LGP Nanogels

In our comprehensive safety assessment of the GOx-LGP-based therapeutic agents, a combination of GOx-LGP and TRAIL-LGP or GOx-DOX-LGP, the hepatic enzyme levels, including AST (aspartate aminotransferase), ALT (alanine aminotransferase), and ALP (alkaline phosphatase), as well as renal function indicators such as BUN (blood urea nitrogen) and CREA (creatinine), was measured. The results showed that these markers remained at baseline levels consistent with those observed in untreated control mice (Figure 8), indicating no significant systemic toxicity. Furthermore, histopathologic examinations of major organs post-treatment revealed no changes, indicating the biocompatibility of these GOx-LGP-based therapeutics. This finding highlights the potential of these GOx-LGP-based therapeutics for clinical application in the treatment of HCC. The use of LGP nanogels as a delivery platform improves both the efficacy and safety of HCC therapies.

**Figure 8:**
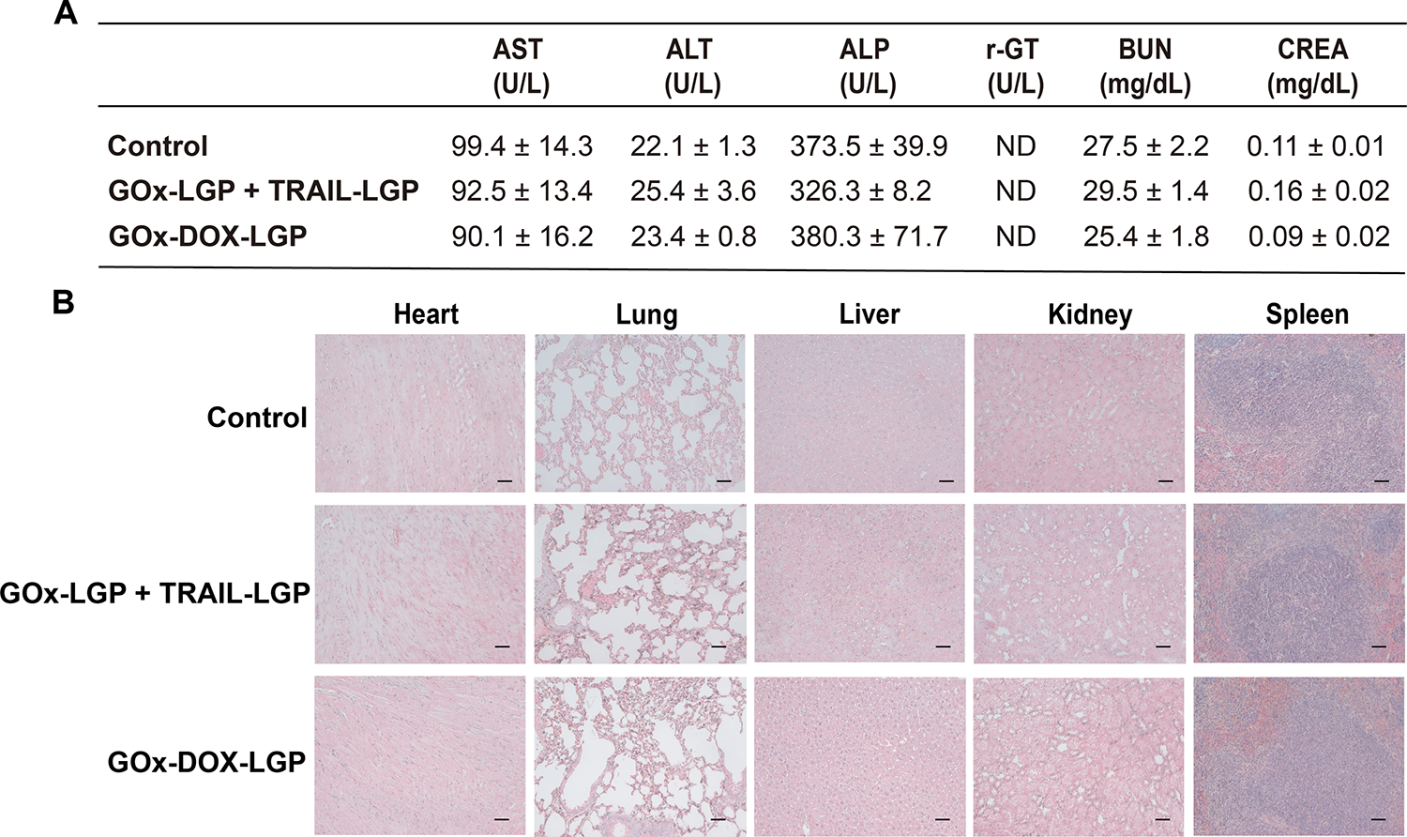
Evaluation of the toxicity profile of the LGP nanogels. (A) Serum was obtained from healthy C3H mice 24 h after IV administration of LGP nanogels to evaluate hepatic and renal toxicity markers, including ALT (alanine aminotransferase), AST (aspartate aminotransferase), γ-GT (gamma-glutamyl transferase), BUN (blood urea nitrogen), and CREA (creatinine) (*n* = 3). Data are the mean values ± S.E.M. (B) Assessment of systemic toxicity via H&E staining revealed no observable histopathological changes in major organs 24 h after IV administration of LGP nanogels. Scale bar: 50 μm.

## 3 Discussion

In this study, we present an LGP nanogel platform engineered for pH-responsive intratumoral delivery of GOx to reprogram glucose metabolism in HCC. By exploiting the acidic tumor microenvironment, the nanogel undergoes controlled destabilization to facilitate localized GOx release, leading to sustained oxidative stress and glycolytic deprivation. This metabolic interference sensitizes tumor cells to apoptosis and significantly enhances the therapeutic efficacy of codelivered agents such as TRAIL and DOX by simultaneously activating intrinsic and extrinsic apoptotic pathways. Moreover, the combination of GOx and DOX induces robust ICD, marked by the release of DAMPs, dendritic cell maturation, and T cell activation, thereby initiating a systemic antitumor immune response. When further combined with immune checkpoint blockade via anti-PD-1-loaded nanogels, this strategy converts the immunosuppressive tumor microenvironment into an immunostimulatory niche, enhancing CD8^+^ T-cell infiltration and promoting long-lasting tumor control in orthotopic HCC models. These findings highlight how tumor metabolic reprogramming, enabled by spatially controlled enzymatic delivery, can overcome therapeutic resistance and trigger potent antitumor immunity in advanced HCC.

The rationale for targeting tumor metabolism in HCC is grounded in its profound dependence on glycolysis metabolism to sustain rapid proliferation and to resist apoptosis. This metabolic adaptation not only supports biosynthesis and redox homeostasis via NADPH production but also protects tumor cells from oxidative stress and stabilizes mitochondrial function.^31^ Clinically, high glycolytic activity—marked by elevated expression of key glycolysis enzymes—is associated with poor prognosis and resistance to chemotherapies, including sorafenib and DOX.^32^ To exploit this vulnerability, our GOx-loaded nanozyme catalyzes glucose oxidation within the tumor microenvironment, inducing ATP depletion and continuous ROS generation. Unlike small-molecule glycolysis inhibitors such as 2-deoxyglucose or LDH blockers, which are often limited by off-target toxicity and compensatory metabolic rewiring, GOx offers sustained, *in situ* glucose consumption and oxidative stress. This sustained metabolic collapse lowers the apoptotic threshold and resensitizes tumor cells to pro-apoptotic agents, amplifying both mitochondrial and death receptor-mediated pathways. These synergistic effects indicate the therapeutic value of enzymatic glycolysis disruption in restoring apoptosis susceptibility in metabolically reprogrammed HCC.^25^

Recently, immunometabolic dysregulation has emerged as a key barrier to effective immunotherapy, as tumors exploit metabolic pathways to suppress antitumor immunity and promote resistance to an immune checkpoint blockade. Beyond fueling proliferation and survival, the glycolytic phenotype of HCC also reinforces immune evasion by creating an immunosuppressive microenvironment. Tumor-derived lactate acidifies the local milieu, impairing dendritic cell maturation and suppressing effector T-cell activity.^33^ In addition to cancer cells, highly glycolytic cancer-associated fibroblasts in the tumor stroma can secrete chemokines to trap T cells and hinder their infiltration, further contributing to immune evasion.^34^ This immunometabolic crosstalk highlights glycolysis as a therapeutic target not only for disrupting tumor metabolism but also for reversing immune suppression. Our pH-sensitive nanozyme enables tumor-specific GOx delivery, initiating glucose depletion and ROS accumulation, which in turn promote ICD through the release of DAMPs such as ATP, HMGB1, and calreticulin. Co-delivery of DOX, a known ICD inducer, further amplifies these immunogenic signals. This dual mechanism transforms immunologically “cold” tumors into “hot” lesions enriched with tumor antigens and pro-inflammatory cues. When combined with anti-PD-1 therapy, the resulting immune activation is sustained through T cell reinvigoration, leading to enhanced CD8^+^ T-cell infiltration and IFN-γ expression. Furthermore, by mitigating lactate-induced T-cell dysfunction, GOx may further support the effector T-cell functionality. Together, the metabolic, apoptotic, and immunological remodeling achieved by the GOx-DOX and anti-PD-1 nanogel combination represents a promising strategy to induce systemic and durable antitumor immunity in HCC.

In addition to its mechanistic advantages, the LGP nanogel platform offers several key translational benefits. It is composed entirely of biodegradable, clinically validated materials—unlike metal-based carriers such as MOFs or ZIFs, which often contain nondegradable frameworks or cytotoxic metal ions.^23,35^ The gelatin-protamine hydrogel core is fully proteolytically degradable, and the PLGA-lipid shell includes FDA-approved components, ensuring excellent biocompatibility. Unlike polymer-enzyme conjugates that require chemical modification, our system enables spontaneous self-assembly and efficient encapsulation of unmodified GOx, preserving enzymatic activity and simplifying production.^24^ The nanogel remains stable in circulation and accumulates in tumors via an EPR effect. Its pH-responsive release is triggered by protonation of acidic residues in gelatin B under mildly acidic conditions (pH ∼ 5.5), which reduces the net negative charge and weakens electrostatic interactions with polycationic protamine, leading to disassembly of the hydrogel matrix and controlled cargo release. We also observed cargo-dependent biodistribution. GOx-LGP accumulated in the spleen, whereas TRAIL-LGP localized in the kidneys, reflecting differences in protein properties. GOx (pI ≈ 4.2, ∼160 kDa) imparted a slightly negative zeta potential (−0.95 mV), while TRAIL (pI ≈ 7.6, ∼20 kDa) yielded a positive potential (+2.84 mV). Early release of free proteins, together with differences in size and charge, further contributed to distinct organ distribution. These results indicate that although the nanogel provides a modular and stable platform, cargo properties critically shape the *in vivo* fate. Accordingly, we administered GOx-LGP and TRAIL-LGP separately rather than constructing a single dual-protein nanogel, enabling independent optimization of dose ratio and schedule and avoiding co-encapsulation constraints within the shared gelatin-protamine core, where disparate size and charge might compete for capacity. Overall, this tunable platform supports co-delivery of proteins and small molecules—including GOx, TRAIL, DOX, and anti-PD-1 antibodies—while maintaining bioactivity, and its modular, tumor-responsive design supports clinical translation for multimodal cancer therapy.

Despite its promise, several challenges remain. Tumor heterogeneity poses a major barrier to uniform efficacy, as not all HCCs are equally glycolytic or immunosuppressive. Some tumors may engage alternative metabolic pathways—such as fatty acid oxidation or glutamine addiction—that confer partial resistance to GOx-induced glucose starvation. Others may harbor intrinsic resistance to TRAIL or DOX due to factors such as p53 mutations or downregulated death receptor expression. These limitations point to the need for a more personalized therapeutic framework. Future applications could incorporate tumor profiling to guide nanogel design, tailoring drug combinations based on metabolic and apoptotic biomarkers—for instance, replacing TRAIL with immunostimulatory cytokines (e.g., IL-12) or alternative checkpoint inhibitors when appropriate. The adaptability of the LGP system supports such customization, enabling rational drug pairing to address the individual tumor vulnerabilities. Clinically, this platform could also augment existing therapies. For example, transarterial chemoembolization (TACE) with DOX-eluting beads is a standard locoregional treatment for intermediate-stage HCC but lacks systemic immune activation and often fails to prevent recurrence. Delivering DOX and GOx via our nanogel could enhance this approach by inducing metabolic collapse and immunogenic cell death, potentially converting a localized treatment into a systemic antitumor strategy. Thus, the integration of metabolic disruption, pro-apoptotic therapy, and immunomodulation within the nanozyme platform provides a versatile and potentially more durable alternative to conventional monotherapies, underscoring the need for continued exploration in biomarker-guided, mechanism-driven combination strategies.

## 4 Conclusions

In summary, this study presents a clinically translatable LGP nanogel platform that enables tumor-selective, pH-responsive delivery of protein and small-molecule therapeutics to achieve immunometabolic reprogramming and overcome therapeutic resistance in HCC. By co-delivering GOx with either TRAIL or DOX, we achieved potent metabolic disruption and amplified apoptotic signaling, resulting in robust tumor suppression in both murine and human orthotopic HCC models. Furthermore, the combination of GOx-DOX-LGP with anti-PD-1 checkpoint blockade transformed the immunosuppressive tumor microenvironment into an immunostimulatory niche, enhancing CD8^+^ T-cell infiltration and cytotoxic activity. Importantly, the LGP nanogels exhibited favorable safety profiles, supporting their translational potential. These findings show the therapeutic promise of targeting tumor metabolism as a central node to synergize apoptosis induction and immune activation, offering a compelling strategy for the effective treatment of advanced HCC.

### 4.1 Methods

Additional materials and methods are included in the Supporting Information (see online Supporting Information and methods).

### 4.2 Materials

Poly­(lactic-*co*-glycolic acid) (PLGA) was purchased from Green Square Materials Inc. (Taiwan). 1,2-dioleoyl-*sn*-glycero-3-phosphocholine (DOPC), 1,2-dioleoyl-3-trimethylammonium-propane (DOTAP), 1,2-dioleoyl-*sn*-glycero-3-phosphate (DOPA), cholesterol, and 1,2-distearoyl-*sn*-glycero-3-phosphoethanolamine-*N*-[amino­(polyethylene glycol)-2000] (DSPE-PEG) were obtained from Avanti Polar Lipids (USA). Bovine serum albumin (BSA) was purchased from MDBio Inc. Albumin from bovine serum (BSA) fluorescein conjugate (FITC-BSA) and the Alexa Fluor 488 Protein Labeling Kit were purchased from ThermoFisher Scientific (USA). Dimethyl sulfoxide (DMSO), glucose oxidase (GOx), protamine sulfate, d-α-Tocopheryl polyethylene glycol 1000 succinate (TPGS), methanol, ethanol, chloroform, cyclohexane, and Igepal-520 were purchased from Sigma-Aldrich (USA). High concentration and phenol-red-free Matrigel and phosphate-buffered saline (PBS) were purchased from Corning (USA). LB broth was obtained from FocusBio (Australia).

### 4.3 Cell Culture

Human HCC cells Hep3B, JHH7, and murine cell line HCA-1 were provided by Dr. Dan Duda (Massachusetts General Hospital, Boston). Hep3B cells were cultured in Minimum Essential Medium Alpha (MEMα) (Sigma-Aldrich, St. Louis, MO), JHH7 cells were cultured in Dulbecco’s Modified Eagle Medium/F12 1:1 (DME/F12) (Hyclone, USA) and HCA-1 cells were cultured in high-glucose Dulbecco’s modified Eagle’s medium (DMEM) (Hyclone, USA). All of the culture medium contained 10% fetal bovine serum (FBS) and 1% antibiotics (penicillin and streptomycin) (Hyclone, Logan, UT). All cell lines were cultured at 37 °C in the incubator (Forma 370, Thermo Fisher Scientific, USA) supplied with 5% CO_2_.

### 4.4 Pathway and Immune Profiling of Candidate Genes Using GSCA and Human Protein Atlas

Pathway activity scores (PAS), pathological stage associations, and immune cell infiltration analyses were performed using the Gene Set Cancer Analysis (GSCA) platform.^36^ To further investigate the expression and localization of candidate genes, data for PGK1, ALDOA, ENO1, and ENO2 were retrieved from the Human Protein Atlas (HPA, https://www.proteinatlas.org). The analysis included RNA expression profiles, immunohistochemistry images, and survival probability data for liver hepatocellular carcinoma (LIHC) tissues. All data were accessed in January 2025.

### 4.5 Glucose Metabolism PCR Array in Human HCC Samples

Tumor and adjacent normal tissues were obtained from HCC patients with IRB approval from Kaohsiung Medical University Hospital (IRB no. KMUHIRB-E­(I)-20240341). Total RNA was extracted using TRIzol Reagent (Invitrogen) according to the manufacturer’s instructions. cDNA was synthesized by using the High-Capacity cDNA Reverse Transcription Kit (Applied Biosystems) on a SimpliAmp Thermal Cycler (Thermo Fisher Scientific). Gene expression profiling was performed using the RT^2^ Profiler PCR Array for Human Glucose Metabolism (PAHS-006ZA, Qiagen). cDNA samples were mixed with a Power SYBR Green PCR Master Mix (Applied Biosystems) and added to array wells preloaded with specific primers. Quantitative PCR was conducted on a QuantStudio 3 Real-Time PCR System (Applied Biosystems). GAPDH was used as an internal control, and relative expression levels in tumor samples were compared to those in matched normal tissues.

### 4.6 Preparation of LGP Nanogels

The cores of LGP, DOTAP-coated gel protein, were first prepared. To prepare a microemulsion (3 mL), 1,2-dioleoyl-3-trimethylammonium-propane (DOTAP) (74 μL, 25 mg/mL) was added to 3 mL of cyclohexane and Igepal-520 (7:3, v/v). Then, the mixture of protein (either 5 μL of 7.5 mg/mL mTRAIL, 5 μL of 4 mg/mL GOx, or 10 μL of 20 mg/mL anti-PD-1 antibody) mixed with protamine (5 μL, 5 mg/mL) and gelatin B (15 μL, 2% w/v) was added to the microemulsion. The emulsion was mixed using a magnetic stir bar for 40 min at room temperature to form solid DOTAP-coated gel protein cores. After that, 3 mL of 100% ethanol was added to disrupt the emulsion. The mixture was centrifuged at 20,133 *g* for 15 min by Centrifuge 5427 R (Eppendorf, Germany). The obtained gel protein cores were washed twice with 100% ethanol to remove the organic solvents and emulsifying agents. Then, the cores were dried under N_2_ and resuspended in 50 μL DMSO by sonication. To formulate the LGP nanogels, an organic phase mixture of 11.5 μL of free lipids (molar ratio = DOPC:DSPE-PEG2000:Cholesterol = 5:3:10), 5 μL of 150 mg/mL PLGA, and 3.75 μL of 100 mg/mL TPGS dissolved in DMSO was mixed with 50 μL of gel protein cores. Then, the whole organic phase was added dropwise into the water phase under sonication by Sonicator Q125 (Qsonica, USA) (setting parameters: duration 100 s; intensity: 30%) (volume ratio of organic phase: water phase = 1:7). The obtained mixture was centrifuged at 15,000 rpm for 30 min, and the nanogels were then resuspended in deionized water for further studies.

### 4.7 Assessment of GOx Catalytic Activity

The enzymatic activity of free GOx and GOx-loaded LGP nanogels (GOx-LGP) was evaluated by using a quantitative peroxide assay. GOx-LGP nanogels containing 0.5 μg/mL (LD) or 1.5 μg/mL (HD) GOx were prepared as previously described. Samples, including free GOx, GOx-LGP, and blank LGP, were each resuspended in 10 μL of deionized water. An additional 10 μL of glucose solution (2 g/L) was added to all groups, except the free GOx-alone control. Subsequently, 200 μL of working reagent from the Pierce Quantitative Peroxide Assay Kit (Pierce Biotechnology, USA) was added to each well. The working reagent consisted of Reagent A (25 mM ammonium ferrous­(II) sulfate in 2.5 M H_2_SO_4_) and Reagent B (100 mM sorbitol, 125 μM xylenol orange in water) mixed at a 1:100 ratio. Absorbance was measured at 595 nm by using a microplate reader to quantify hydrogen peroxide production as an indicator of catalytic activity.

### 4.8 HCC Orthotopic Animal Model

C3H/HeNCrNarl male mice and Nude male mice were purchased from the National Laboratory Animal Center (Taipei, Taiwan). All animals received humane care in compliance with the Guide for the Care and Use of Laboratory Animals published by the National Academy of Sciences, and all study procedures and protocols were approved by the Animal Research Committee of National Tsinghua University. Murine HCC HCA-1 cells were implanted orthotopically in the livers of 6–8 week old C3H/HeNCrNarl male mice. Human HCC JHH-7 cells were orthotopically implanted in the liver of 6–8 week old male nude mice. Particularly, 1 × 10^6^ cells (prepared in 20 μL of a 1:1 solution of Matrigel in PBS) were injected in the subcapsular region of the liver using a 28G needle. Two weeks after the first treatment, mice were sacrificed for further analysis. The tumor volume was measured after 2 weeks of treatment and calculated by the formula: volume = (width × length × height)/2. The primary tumor tissue and distal lung metastatic tumor tissue were collected for further analysis. The sample size was determined based on power calculations to detect statistically significant differences with at least 80% power and a significance level (α) of 0.05, assuming an effect size of 0.75 and a type I error of 0.05. All animals were randomly assigned to experimental groups to minimize selection bias and ensure a balanced allocation across treatment arms.

### 4.9 TRAIL and GOx Combination Treatment

To evaluate the anticancer efficacy, mTRAIL-LGP (3.5 mg/kg, three doses per week) and GOx-LGP (0.4 mg/kg, three doses per week) were intravenously administered to HCA-1 tumor-bearing mice starting from 10 days after the implantation. For the human HCC model, the JHH7-tumor-bearing mice were treated with hTRAIL-LGP (2.5 mg/kg, three doses per week) and GOx-LGP (0.4 mg/kg, three doses per week) starting from 5 days after implantation.

### 4.10 GOx-DOX-LGP and aPD1-LGP Combination Treatment

GOx-DOX-LGP (loaded with 0.4 mg/kg GOx and 1.8 mg/kg DOX, three doses per week) was intravenously administered to HCA-1 tumor-bearing mice starting from 10 days after the implantation alone or in combination with aPD1-LGP (4 mg/kg, three doses per week). The tumor tissues were collected for further analysis.

### 4.11 Statistics

Statistical analyses were performed using GraphPad Prism 6. Data analyses were calculated using the Student’s *t* test. Two-way ANOVA followed by a multiple comparison was used to compare data sets between 2 and 3 groups. A *p*-value < 0.05 were considered statistically significant for all tests.

## Supplementary Material



## Data Availability

The RNA-seq data set has been submitted to the Gene Expression Omnibus (GEO) and is available under accession number GSE295520. All other data are available in this manuscript, Supporting Information, or will be made available upon reasonable request.

## Notes

Supplementary Method and Materials; the mRNA expression levels of 84 key genes involved in glucose metabolism were analyzed in HCC patient samples using the RT^2^ Profiler PCR Array (Table S1); protein loading efficiency of LGP-based nanogel (Table S2); GOBP pathway sets revealed showed downregulation of glucose and fatty acid metabolic pathways, alongside marked upregulation of immune-related in murine HCA-1 tumors among GOx-DOX-LGP treatment relative to Control (Table S3); flow cytometry analysis shows an increase of intracellular ROS levels in HCA-1 cells following a 3 h treatment with GOx, measured by H_2_DCFDA fluorescence (Figure S1); Bliss synergy scores for interaction of GOx in combination with TRAIL or DOX (Figure S2); representative TEM images of LGP-based nanogels (Figure S3); tumor volume changes in response to TRAIL-LGP and GOx-LGP nanogels (Figure S4); representative images of tumor from orthotopic human JHH7 xenograft mice in response to either GOx-LGP, TRAIL-LGP or both in combination (Figure S5); gating strategy for flow cytometry analysis of granzyme B^+^, IFN-γ^+^, and Ki67^+^ cytotoxic CD8^+^ T cells in orthotopic HCC tumors following treatment with different formulations (Figure S6); increased splenic CD44^+^CD62L^+^ central memory T cells in GOx-DOX-LGP-treated HCC mice, as determined by flow cytometry (Figure S7); tumor volume changes in response to GOx-DOX-LGP in combine with aPD1-LGP (Figure S8); Representative images of murine orthotopic HCC tumors in response to either GOx-DOX-LGP or aPD-1-LGP for single treatment groups, and both nanogels for combination treatment (Figure S9) (PDF)

## Abbreviations

HCC: hepatocellular carcinoma
NK: natural Killer
GOx: glucose oxidase
LGP: lipid–gelatin–protamine
PLGA: poly­(lactic-*co*-glycolic acid)
EPR: enhanced permeability and retention effect
DOX: doxorubicin
TRAIL: tumor necrosis factor-related apoptosis-inducing ligand
ICIs: immune checkpoint inhibitors
PD-1: programmed cell death protein 1
ROS: reactive oxygen species
ER: endoplasmic reticulum
DR4: death receptor 4
DR5: death receptor 5
ICD: immunogenic cell death
DAMP: damage-associated molecular patterns
TCGA-LIHC: The Cancer Genome Atlas- Liver Hepatocellular Carcinoma
PGK1: phosphoglycerate kinase 1
ALDOA: aldolase A
ENO1: enolase 1
qRT-PCR: quantitative real-time reverse transcription polymerase chain reaction
ATF-4: activating transcription factor 4
CHOP: C/EBP homologous protein
DOTAP: 1,2-dioleoyl-3-trimethylammonium propane
SEM: scanning electron microscopy
TUNEL: terminal deoxynucleotidyl transferase dUTP nick end labeling.
