# Supplementary material for: Stimuli-Responsive Nanozyme Reprograms Tumor Immunometabolism and Overcomes Therapeutic Resistance in Hepatocellular Carcinoma
Source: ACS Nano. 2026 Jan 6;20(2):1870–84. doi: 10.1021/acsnano.5c11352 (PMC12825357; doi:10.1021/acsnano.5c11352)
Supplement: Supplementary file 1 [file nn5c11352_si_001.pdf]

Supporting Information

Stimuli-Responsive Nanozyme Reprograms Tumor Immunometabolism and Overcomes Therapeutic Resistance in Hepatocellular Carcinoma

Yen-Nhi Ngoc Ta[1,2], Van-Anh Thi Nguyen[1,2], Thu-Thuy Can[1], Meng-Cheng Hsieh[1], Bang Giang Thi Cao[1], Dehui Wan[1], Chian-Hui Lai[3], Chun-Chieh Wu[4], Fu-Fei Hsu[5], Yu-Ting Yen[6], Shen-Nien Wang[7,8,]* & Yunching Chen[1,9]**

[1] Institute of Biomedical Engineering, National Tsing Hua University, Hsinchu 30013, Taiwan

[2] International Intercollegiate PhD Program, National Tsing Hua University, Hsinchu 30013, Taiwan

[3] Graduate Institute of Biomedical Engineering, National Chung Hsing University, Taichung 40227, Taiwan

[4] Department of Pathology, Kaohsiung Medical University Hospital, Kaohsiung 80708, Taiwan

[5] Institute of Biomedical Sciences, Academia Sinica, Taipei 11529, Taiwan

[6] Institute of Translational Medicine and New Drug Development, School of Medicine, China Medical University, Taichung 40402, Taiwan

[7] College of Medicine, Kaohsiung Medical University, Kaohsiung 80708, Taiwan

[8] Pingtung Hospital, Ministry of Health and Welfare, Pingtung 900, Taiwan

[9] Department of Chemistry, National Tsing Hua University, Hsinchu 30013, Taiwan

Supplementary Method and Materials

Real-time PCR of HCC patient sample

cDNA derived from HCC patient tumor and adjacent normal tissues was further analyzed by quantitative PCR using SYBR Green Real-Time PCR Master Mix (Applied Biosystems) on a QuantStudio 3 Real-Time PCR System (Applied Biosystems). Gene expression was normalized to GAPDH as an internal control, and relative expression levels in tumor samples were compared to matched normal tissues. Primer sequences are listed below:

Primer	Forward sequence (5'-3')	Reverse sequence (5'-3')
hGADPH	AATCCCATCACCATCTTCCA	TGGACTCCACGACGTACTCA
hALDOA	GTTATCAAATCCAAGGGCGGTGTT	AGTCAGCTCCGTCCTTCTTGTAC
hPGK1	CAAGGTTAAAGCCGAGCCAGCCAA	GCCTTCTGTGGCAGATTGACTCC
hENO1	GCTCCGGGACAATGATAAGACTCG	CTGTTCCATCCATCTCGATCATC
hENO2	TGAAGGCAGTGGACCACATCAACT	AGAGACACACCCAGGATGGCATT

Detection of intracellular ROS

HCA-1 and Hep3B cells were collected at a density of 6×10^5 cells/mL (1 mL per tube) and washed with PBS. Cells were stained with 25 μM H_2DCFDA (Sigma) in 0.5 mL PBS for 30 minutes at 37 °C in the dark. After centrifugation at 3000 rpm for 5 minutes, cells were resuspended in 1 mL glucose-free DMEM (Gibco) supplemented with 10 g/L D-glucose and glucose oxidase (GOx) at 0, 1, or 2 ng/mL, and incubated for 3 hours at 37 °C. Cells were then centrifuged, resuspended in 500 μL PBS, filtered through a 40 μm cell strainer, and analyzed by flow cytometry.

Characterization of LGP nanogels

The particles size and surface charge of the particles formulated as describe above were examined using a Zetasizer (3000HS, Malvern Instruments Ltd., UK) at room temperature. The protein encapsulation efficiency of nanogels was determined via Alexa Fluor 488-labelled proteins (TRAIL, GOx or anti-PD-1 antibody) fluorescent intensity measured by Spark Multimode Microplate Reader (Tecan, Switzerland). Briefly, fluorescent protein and blank were encapsulated in nanogels simultaneously as above protocol, then the supernatant solutions in each centrifugation steps were collected to measure the fluorescence signal of unencapsulated protein. Finally, the encapsulation efficiency was calculated by the standard curve of serial dilution of free fluorescent proteins in blank supernatant solutions, which subtracts the effect of background on the signal.

The protein loading efficiency was calculated as the ratio of the encapsulated protein to the total weight of nanoparticles.

Evaluation of pH-sensitive release kinetics of protein-loaded LGP nanogels

The pH-responsive release of protein from LGP nanogels was evaluated in phosphate buffers at pH 5.5, 6.5, and 7.4. FITC-BSA-loaded nanogels were evenly distributed into Eppendorf tubes and suspended in 1 mL of the corresponding buffer. The samples were incubated at 37 °C in an orbital shaker at 100 rpm. At predetermined time points, the nanogels were collected by centrifugation at 15,000 rpm for 30 minutes at 4 °C. The resulting pellets were resuspended in 100 μL of pH 7.4 buffer, and fluorescence intensity was measured to quantify protein release.

Expression and purification of His_6-tagged TRAIL protein

A His_6-tagged TRAIL expression plasmid was constructed as previously described [1]. For protein expression, the plasmid was transformed into Escherichia coli BL21 (DE3) cells. An overnight bacterial culture was diluted 1:100 into fresh LB medium (25 g/L) and incubated at 37 °C with shaking at 200 rpm until the optical density at 600 nm (OD_{600}) reached 0.6–0.8. Protein expression was induced by adding 0.5 mM isopropyl-β-D-thiogalactopyranoside (IPTG), followed by incubation at 30 °C with shaking for 3 hours.

Bacterial pellets were harvested and lysed by sonication in lysis buffer containing 50 mM sodium phosphate (pH 8.0), 300 mM NaCl, 10 mM imidazole, and 10 mM β-mercaptoethanol. The lysate was clarified by centrifugation, and the supernatant was loaded onto a gravity-flow column packed with Ni-NTA agarose beads. After washing with lysis buffer containing 20 mM imidazole, the bound His_6-TRAIL protein was eluted using buffer containing 250 mM imidazole and subsequently dialyzed against PBS containing 10 mM β-mercaptoethanol. Protein concentration was measured using a NanoDrop 1000 spectrophotometer.

Cell viability assay

Cell viability was assessed using the MTT assay. Hep3B and HCA-1 (1,000 cells/well) cells were seeded in 96-well plates and cultured for 24 hours. Cells were then treated with glucose oxidase (GOx) in combination with either TRAIL or doxorubicin at varying concentrations. After 24 hours of treatment, 15 μL of MTT solution (5 mg/mL in PBS) was added to each well, followed by a 3-hour incubation at 37 °C. The medium was then removed, and 50 μL of DMSO was added to

dissolve the resulting formazan crystals. Absorbance was measured at 570 nm using a microplate reader (Multiskan GO, Thermo Fisher Scientific, USA).

Calculation of synergy

The synergistic interaction between GOx and TRAIL (or DOX) was evaluated using the Bliss independence model[2]. The expected fractional inhibition for the combination was calculated as:

$$E_{Bliss} = E_A + E_B - (E_A \times E_B)$$

The **Synergy Coefficient (SC)** was determined by the ratio:

$$SC = \frac{E_{AB}}{E_{Bliss}}$$

Where

$$E = 1 - \left(\frac{\text{Viability}}{100}\right)$$

E_A : fractional inhibition by drug A

E_B : fractional inhibition by drug B

E_{AB} : fractional inhibition by combination

An **SC > 1** indicates synergistic interaction, **SC = 1** represents an additive effect, and **SC < 1** suggests antagonism.

Assessment of apoptosis by flow cytometry

HCA-1 and Hep3B cells (5×10^5 cells/well) were seeded in 12-well plates and cultured for 24 hours. Cells were then treated with either free drugs (TRAIL, GOx, or doxorubicin) or their corresponding LGP nanogel formulations (TRAIL-LGP, GOx-LGP, or DOX-LGP) (mTRAIL: 10 µg/mL; hTRAIL: 1µg/mL; GOx: 4 ng/mL; DOX: 0.5 µM) . After 24 hours of treatment, cells were harvested, washed twice with cold PBS, and stained with Annexin V according to the manufacturer's instructions (BD Biosciences, CA, USA). Apoptotic cell populations were analyzed using a BD FACSAria III flow cytometer (Becton Dickinson, CA, USA), and the data were processed with FACSDiva™ software.

Quantitative real-time PCR analysis *in vitro* and *in vivo*

In vitro, HCA-1 cells were seeded at a density of 5×10^4 cells/well in 12-well plates, cultured for 24 hours, and treated with GOx-LGP for an additional 24 hours. Cells were then washed with 1 mL of PBS to remove residual drug, and total RNA was extracted using 400 µL of TRIzol™ Reagent (Invitrogen, Cat#15596018, USA) according to the manufacturer's instructions.

In vivo, orthotopic HCC were established by injecting 1×10^6 HCA-1 cells (in 20 μL of a 1:1 Matrigel/PBS mixture) into the subcapsular region of the liver in 6–8-week-old male C3H/HeNCrNarl mice. Mice were intravenously administered GOx-DOX-LGP (containing 0.4 mg/kg GOX and 1.8 mg/kg DOX) on days 18, 19, and 20 post-implantation. Tumor tissues were harvested at day 21, and total RNA was isolated using the RNeasy RNA Purification Kit (Qiagen, USA) following the manufacturer's protocol.

Isolated RNA was reverse transcribed into cDNA using the High-Capacity cDNA Reverse Transcription Kit (Applied Biosystems, USA) on a SimpliAmp™ Thermal Cycler (Thermo Fisher Scientific, MA, USA). Quantitative PCR was performed with SYBR™ Green Master Mix (Applied Biosystems, USA) on a QuantStudio™ 3 Real-Time PCR System (Applied Biosystems, USA). Gene expression levels were calculated using the comparative Ct (ΔΔCt) method and normalized to GAPDH as the internal control. Primer sequences used are listed below.

Primer	Forward sequence (5'-3')	Reverse sequence (5'-3')
mGADPH	CTGCCACCCAGAAGACTGTG	GGTCCTCAGTGTAGCCCAAG
mHMBG1	GGCTGACAAGGCTCGTTATG	GGGCGGTACTCAGAACAGAAC
mCRT	AAGAGCAGT TCTTGGACGGA	CACCAGTGTCTGGCCCTTAT
mATF-4	CCGGAAATTCGTCAACGAGC	AGATCGTCCTAAAGGCCCCA
CHOP	CCTGAGGAGAGAGTGTTCCAG	GACACCGTCTCCAAGGTGAA
mDR5	AAAACGGCTTGGGCATCTTGGC	AGACGGTTCCAGGAGTCAAAGG
mIFN-α	TGTCTGATGCAGCAGGTGG	AAGACAGGGCTCTCCAGAC
mIFN-β	ATGGTGGTCCGAGCAGAGAT	CCACCACTCATTCTGAGGCA
mIFN-γ	CAGCAACAGCAAGGCGAAAAAGG	TTTCCGCTTCCTGAGGCTGGAT
CXCL9	AGTGTGGAGTTCGAGGAACC	GAGTCCGGATCTAGGCAGG
mCXCL10	CCAAGTGCTGCCGTCATTTTC	GGCTCGCAGGGATGATTTCAA
mCXCL11	AGCTGCTCAAGGCTTCCTTA	AGTAACAATCACTTCAACTTTGTCG

Next-generation sequencing gene expression analysis

RNA integrity from the treatment and control samples, extracted using the RNeasy Kit (Qiagen), was assessed with the RNA Nano6000 assay kit (Agilent Technologies, CA, USA). Library preparation and sequencing were performed by Biotools Co., Ltd. The sequencing data (FASTQ

files) were aligned to the reference genome using TopHat v2.0.12. HTSeq v0.6.1 was used to quantify gene-level read counts, and FPKM values were calculated based on gene length and the number of mapped reads.

Gene expression levels were determined based on read counts derived from RNA sequencing data; specifically, the read counts obtained through alignment analysis were used to quantify gene expression. For relative gene expression analysis, normalization was performed using edgeR (v3.28.1), and differential expression analysis was conducted with DESeq2 (v1.26.0) [3, 4]. Genes exhibiting significant changes in expression were identified based on p-values and a false discovery rate (FDR) threshold of <0.05 following edgeR analysis. Differentially expressed gene patterns were visualized using the ClustVis web tool, while Gene Set Enrichment Analysis (GSEA) was used to explore associated biological pathways and variability [5, 6]. The dataset has been submitted to the Gene Expression Omnibus (GEO) and is available under accession number GSE295520.

Western blot analysis

HCA-1 cells and Hep3B cells (5×10^5 cells per well) were seeded in 12-well plates, cultured for 24 hours, and then treated with GOx or GOx-LGP for an additional 24 hours. Following treatment, cells were washed with PBS and lysed in radioimmunoprecipitation assay (RIPA) buffer on ice for 10 minutes. The lysates were centrifuged at 12,000 rcf to collect the protein supernatant, which was then mixed with 4X Laemmli Sample Buffer (Bio-Rad, Hercules, CA) and heated at 95°C for 5 minutes. Proteins were separated on a 10% bis-acrylamide gel and subsequently transferred to a PVDF membrane. Membranes were blocked in 5% skim milk in PBS for 1h and then incubated overnight with primary antibodies against DR5 (ab8416, Abcam), ATF4 (D4B8), CHOP (L63F7) (Cell Signaling, Danvers, MA) and β-actin (Sigma-Aldrich, St. Louis, MO) at 4°C. After three 3-minute washes with PBST (0.1% Tween 20 in PBS), the membranes were incubated with HRP-conjugated secondary antibodies (Antibodies Inc., Davis, CA) for 1 hour at room temperature. The autoradiography was conducted after ECL reagent (Thermo Scientific, Rockford, IL) was added to the membranes.

Biodistribution study

Murine HCA-1 cells were orthotopically implanted into the livers of 6–8-week-old male C3H/HeNCrNarl mice. On day 24 post-implantation, mice were intravenously injected with either

Alexa488-labeled protein loaded LGP nanogels or free-form Alexa488-labeled protein. The administered protein was either mTRAIL (3.5 mg/kg) or GOx (0.4 mg/kg). Four hours post-injection, mice were euthanized and major tissues were harvested. Tissues were homogenized in lysis buffer containing 0.1% SDS, 1% Triton X-100, 0.1% sodium deoxycholate, 10 mM Tris-HCl, and 140 mM NaCl. The lysates were used to quantify fluorescence intensity, and protein accumulation was calculated by referencing a standard curve generated from known concentrations of proteins.

Pharmacokinetic study

Male C3H/HeNCrNarl mice were intravenously injected with either Alexa488-labeled protein-loaded LGP nanogels or free-form Alexa488-labeled protein. The administered proteins included mTRAIL (3.5 mg/kg) or GOx (0.4 mg/kg). At designated time points, 200 μL of blood was collected and immediately mixed with 20 μL of 0.5 μM EDTA in PBS to prevent coagulation. The fluorescence intensity of Alexa Fluor 488-labeled proteins in plasma was measured using a microplate reader (Spark 10M, Tecan, Germany). Protein concentrations were quantified based on a standard curve generated from known concentrations of proteins.

Flow cytometry analysis

HCA-1-tumor-bearing mice were perfused through intracardiac injection of 30 mL of saline. Tumor tissues were collected and digested at 37 °C for 30 minutes with collagenase type 1A (1.5 mg/mL) and hyaluronidase (1.5 mg/mL) in serum-contained RPMI culture medium. A 40 μm strainer was used to filter the enzyme-digested tumor tissues. Cell suspensions were washed and resuspended in 1% BSA (in PBS), following by staining with antibodies. To assess the expression of specific markers in intratumoral $CD8^+$ T cells, cells were first washed and fixed in 4% paraformaldehyde at 4 °C for 15 minutes, then stored overnight at 4 °C in the dark in PBS containing 1% BSA. The cells were subsequently permeabilized using Cytofix/Cytoperm solution (BD Biosciences, CA, USA) and stained with antibodies against intracellular markers, following the manufacturer's protocol.

Regarding memory T cells evaluation, spleen from HCA-1-tumor-bearing mice were collected after perfusion and homogenized in serum-containing RPMI-1640 medium. The cell suspensions were centrifuged, and red blood cells were lysed with ACK buffer for 5 minutes before neutralization with RPMI. After two washes with serum-containing RPMI, the cells were resuspended, filtered through 70 μm strainer. Cell suspensions were washed and resuspended in

1% BSA (in PBS), following by filtering by a 40 μm stariner and staining with antibodies for 20 minutes on ice, protected from light. Cells were then washed, resuspended in 1% BSA, and analyzed by flow cytometry.

Flow cytometry was performed using a BD FACSAria III flow cytometer (Becton Dickinson, CA, USA), and data were analyzed using FACSDiva™ software.The following antibodies were used for flow cytometry analysis: CD3e-APC (no. 145-2C11), CD8-PE-Cy7 (no. 53-6.7), CD4-PE (no. RM4-5), CD44-PE (no. 561860), CD62L-FITC (no. 561917), CD16/CD32 BD Fc Block (no. 2.4G2), IFN-γ-APC-Cy7 (no. XMG1.2) and 7-AAD, all from BD Biosciences; granzyme B-FITC (no. GB11) from Biolegend; and IFN-alpha (no.22100- 3) from R&D Systems, USA.

Assessment of apoptosis by TUNEL staining

Frozen sections of murine HCC tumor tissues (HCA-1) and Paraffin-embedded tissue sections of human HCC tumor tissues (JHH7) were stained by using a DeadEnd™ Fluorometric TUNEL System (Promega, USA) and DeadEnd™ Colorimetric TUNEL System (Promega, USA), respectively, according to the manufacturer's guideline. The apoptotic index was calculated as the fraction of apoptotic nuclei relative to DAPI-stained cell nuclei.

Table S1. The mRNA expression levels of 84 key genes involved in glucose metabolism were analyzed in HCC patient samples using the RT² Profiler PCR Array. Data are presented as fold changes relative to the corresponding levels in adjacent normal tissue samples (n=3)

Gene symbol	Gene Bank	Description	Fold regulation
**Glycolysis**			
ALDOA	NM_000034	Aldolase A, fructose-bisphosphate	4.505
ALDOB	NM_000035	Aldolase B, fructose-bisphosphate	29.616
ALDOC	NM_005165	Aldolase C, fructose-bisphosphate	2.356
BPGM	NM_001724	2,3-bisphosphoglycerate mutase	8.002
ENO1	NM_001428	Enolase 1, (alpha)	4.099
ENO2	NM_001975	Enolase 2 (gamma, neuronal)	3.626
ENO3	NM_001976	Enolase 3 (beta, muscle)	5.939
GALM	NM_138801	Galactose mutarotase (aldose 1-epimerase)	5.041
GCK	NM_000162	Glucokinase (hexokinase 4)	4.384
GPI	NM_000175	Glucose-6-phosphate isomerase	28.285
HK2	NM_000189	Hexokinase 2	4.743
HK3	NM_002115	Hexokinase 3 (white cell)	4.156
PFKL	NM_002626	Phosphofructokinase, liver	4.712
PGAM2	NM_000290	Phosphoglycerate mutase 2 (muscle)	4.767
PGK1	NM_000291	Phosphoglycerate kinase 1	6.400
PGK2	NM_138733	Phosphoglycerate kinase 2	8.207
PGM1	NM_138733	Phosphoglucomutase 1	7.815
PGM2	NM_018290	Phosphoglucomutase 2	6.422
PGM3	NM_015599	Phosphoglucomutase 3	9.675
PKLR	NM_000298	Phosphoglucomutase 1	6.130
TPI1	NM_000365	Triosephosphate isomerase 1	5.207
**Gluconeogenesis**			
FBP1	NM_000507	Fructose-1,6-bisphosphatase 1	114.312
FBP2	NM_003837	Fructose-1,6-bisphosphatase 2	3.985
G6PC	NM_003837	Glucose-6-phosphatase, catalytic subunit	8.054
G6PC3	NM_138387	Glucose 6 phosphatase, catalytic, 3	3.327
PC	NM_000920	Pyruvate carboxylase	4.451
PCK1	NM_002591	Phosphoenolpyruvate carboxykinase 1 (soluble)	19.668
PCK2	NM_004563	Phosphoenolpyruvate carboxykinase 2 (mitochondrial)	3.937
**Regulation of Glucose metabolism**			
PDK1	NM_002610	Pyruvate dehydrogenase kinase, isozyme 1	6.765
PDK2	NM_002611	Pyruvate dehydrogenase kinase, isozyme 2	3.016
PDK3	NM_005391	Pyruvate dehydrogenase kinase, isozyme 3	5.637

Gene symbol	Gene Bank	Description	Fold regulation
PDK4	NM_002612	Pyruvate dehydrogenase kinase, isozyme 4	8.168
PDP2	NM_020786	Pyruvate dehydrogenase phosphatase catalytic subunit 2	3.700
PDPR	NM_017990	Pyruvate dehydrogenase phosphatase regulatory subunit	3.797
**Pentose Phosphate Pathway**			
G6PD	NM_000402	Glucose-6-phosphate dehydrogenase	4.441
H6PD	NM_004285	Hexose-6-phosphate dehydrogenase (glucose 1-dehydrogenase)	2.943
PGLS	NM_012088	6-phosphogluconolactonase	6.594
PRPS1	NM_002764	Phosphoribosyl pyrophosphate synthetase 1	17.194
PRPS1L1	NM_175886	Phosphoribosyl pyrophosphate synthetase 1-like 1	7.718
PRPS2	NM_002765	Phosphoribosyl pyrophosphate synthetase 2	4.218
RBKS	NM_022128	Ribokinase	10.998
RPE	NM_199229	Ribulose-5-phosphate-3-epimerase	8.026
RPIA	NM_144563	Ribose 5-phosphate isomerase A	5.740
TALDO1	NM_006755	Transaldolase 1	47.055
TKT	NM_001064	Transketolase	30.598
**Tricarboxylic Acid (TCA) Cycle**			
ACLY	NM_001096	ATP citrate lyase	2.294
ACO1	NM_002197	Aconitase 1, soluble	3.085
ACO2	NM_001098	Aconitase 2, mitochondrial	3.424
CS	NM_004077	Citrate synthase	3.778
DLAT	NM_004077	Dihydrolipoamide S-acetyltransferase	4.563
DLD	NM_000108	Dihydrolipoamide dehydrogenase	6.421
DLST	NM_000108	Dihydrolipoamide S-succinyltransferase (E2 component of 2-oxo-glutarate complex)	3.403
FH	NM_000143	Fumarate hydratase	169.962
IDH1	NM_005896	Isocitrate dehydrogenase 1 (NADP+), soluble	4.395
IDH2	NM_002168	Isocitrate dehydrogenase 2 (NADP+), mitochondrial	2.503
IDH3A	NM_002168	Isocitrate dehydrogenase 3 (NAD+) alpha	4.403
IDH3B	NM_174856	Isocitrate dehydrogenase 3 (NAD+) beta	3.708
IDH3G	NM_174869	Isocitrate dehydrogenase 3 (NAD+) gamma	3.180
MDH1	NM_005917	Malate dehydrogenase 1, NAD (soluble)	5.693
MDH1B	NM_001039845	Malate dehydrogenase 1B, NAD (soluble)	8.511
MDH2	NM_005918	Malate dehydrogenase 2, NAD (mitochondrial)	5.553
OGDH	NM_002541	Oxoglutarate (alpha-ketoglutarate) dehydrogenase (lipoamide)	3.303
PC	NM_000920	Pyruvate carboxylase	3.338
PCK1	NM_002591	Phosphoenolpyruvate carboxykinase 1 (soluble)	14.751
PCK2	NM_004563	Phosphoenolpyruvate carboxykinase 2 (mitochondrial)	2.953
PDHA1	NM_000284	Pyruvate dehydrogenase (lipoamide) alpha 1	63.428

Gene symbol	Gene Bank	Description	Fold regulation
PDHB	NM_000925	Pyruvate dehydrogenase (lipoamide) beta	10.856
SDHA	NM_004168	Succinate dehydrogenase complex, subunit A, flavoprotein (Fp)	3.630
SDHB	NM_003000	Succinate dehydrogenase complex, subunit B, iron sulfur (Ip)	5.862
SDHC	NM_003001	Succinate dehydrogenase complex, subunit C, integral membrane protein, 15kD	8.173
SDHD	NM_003001	Succinate dehydrogenase complex, subunit D, integral membrane protein	5.247
SUCLA2	NM_003850	Succinate-CoA ligase, ADP-forming, beta subunit	5.183
SUCLG1	NM_003849	Succinate-CoA ligase, alpha subunit	47.155
SUCLG2	NM_003848	Succinate-CoA ligase, GDP-forming, beta subunit	27.645
**Glycogen Synthesis**			
GBE1	NM_000158	Glucan (1,4-alpha-), branching enzyme 1	12.305
GYS1	NM_002103	Glycogen synthase 1 (muscle)	3.477
GYS2	NM_021957	Glycogen synthase 2 (liver)	5.884
UGP2	NM_006759	UDP-glucose pyrophosphorylase 2	6.180
**Regulation of Glycogen Metabolism**			
GSK3A	NM_019884	Glycogen synthase kinase 3 alpha	2.871
GSK3B	NM_002093	Glycogen synthase kinase 3 beta	7.799
PHKA1	NM_002637	Phosphorylase kinase, alpha 1 (muscle)	5.048
PHKB	NM_000293	Phosphorylase kinase, beta	6.804
PHKG1	NM_006213	Phosphorylase kinase, gamma 1 (muscle)	3.660
PHKG2	NM_000294	Phosphorylase kinase, gamma 2 (testis)	3.186
**Glycogen degradation**			
AGL	NM_000028	Amylo-alpha-1, 6-glucosidase, 4-alpha-glucanotransferase	8.929
PGM1	NM_002633	Phosphoglucomutase 1	7.815
PGM2	NM_018290	Phosphoglucomutase 2	6.422
PGM3	NM_015599	Phosphoglucomutase 3	9.675
PYGL	NM_002863	Phosphorylase, glycogen, liver	16.711
PYGM	NM_005609	Phosphorylase, glycogen, muscle	6.076

Table S2. Protein loading efficiency of LGP-based nanogel (n = 3).
Data are presented as mean ± S.D.

Protein (LGP)	Protein loading efficiency (%)
**GOx** (GOx-LGP)	3.4 ± 0.2
**TRAIL** (TRAIL-LGP)	6.6 ± 1.0
**Anti-PD-1 antibody** (aPD1-LGP)	26.6± 2.5

Table S3. GOBP pathway sets revealed showed downregulation of glucose and fatty acid metabolic pathways, alongside marked upregulation of immune-related in murine HCA-1 tumors among GOx-DOX-LGP treatment relative to Control (n = 3).

Name of gene set	ES	NES	P value
**Fatty acid metabolic process**	-0.757	-1.888	< 0.001
**Amino acid metabolic process**	-0.735	-1.818	< 0.001
**Tryptophan metabolic process**	-0.857	-1.614	0.001
**Glucose metabolic process**	-0.621	-1.532	< 0.001
**Glutamine metabolic process**	-0.636	-1.373	0.068
**Carbohydrate metabolic process**	-0.539	-1.347	< 0.001
**Granzyme mediated programmed cell death signaling pathway**	0.920	2.545	0.005
**T helper 1 type immune response**	0.575	2.078	0.024
**Response to interferon beta**	0.518	2.073	0.056
**Regulation of T cell receptor signaling pathway**	0.418	1.687	0.048
**Neutrophil activation**	0.464	1.651	0.014
**Regulation of programmed necrotic cell death**	0.459	1.489	0.074
**Leukocyte activation involved in inflammatory response**	0.399	1.448	0.054
**Granulocyte activation**	0.382	1.395	0.036
**Positive regulation of natural killer cell mediated immunity**	0.373	1.357	0.058

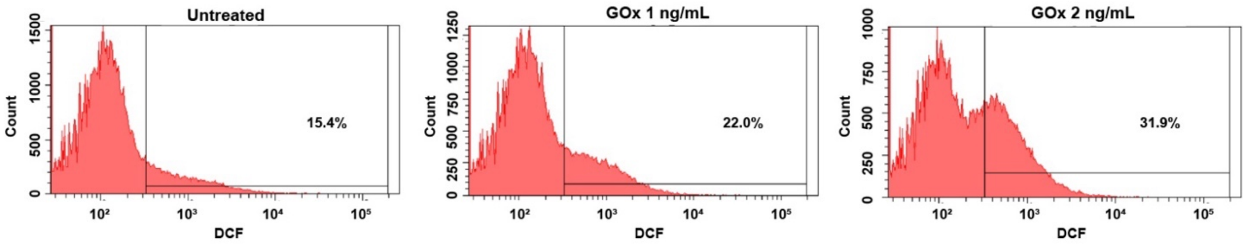

Figure S1. Flow cytometry analysis shows an increase of intracellular reactive oxygen species (ROS) levels in murine (HCA-1) following a 3-hour treatment with GOx, measured by H_2DCFDA fluorescence. Representative plots from individual samples are shown.

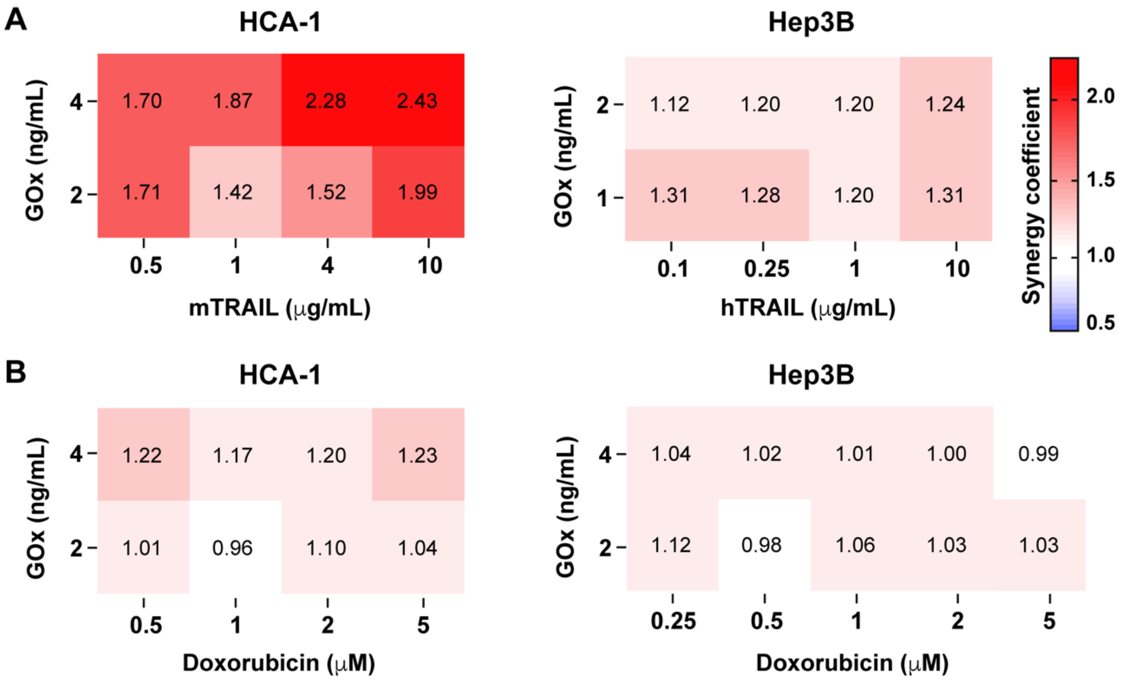

Figure S2. Bliss synergy scores for interaction of GOx in combination with TRAIL (A) or doxorubicin (DOX) (B) (n=6). Synergy scores of less than 1, equal to 1 and larger than 1 are interpreted as antagonistic, addictive and synergistic effects, respectively. Data are the mean values ± S.E.M.

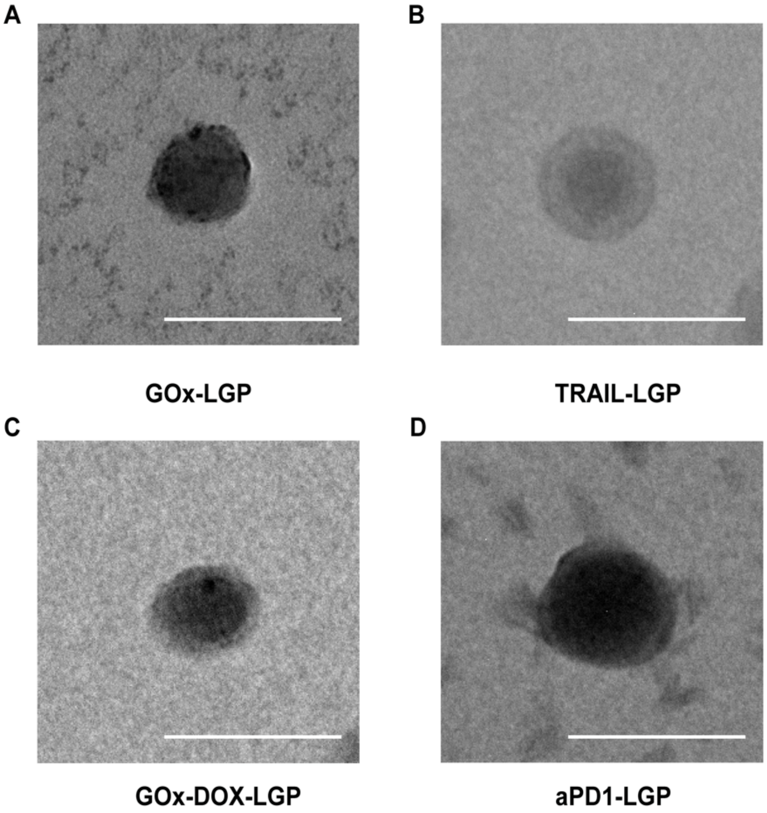

Figure S3. Representative TEM images (scale bar: 200 nm) of LGP-based nanogels.

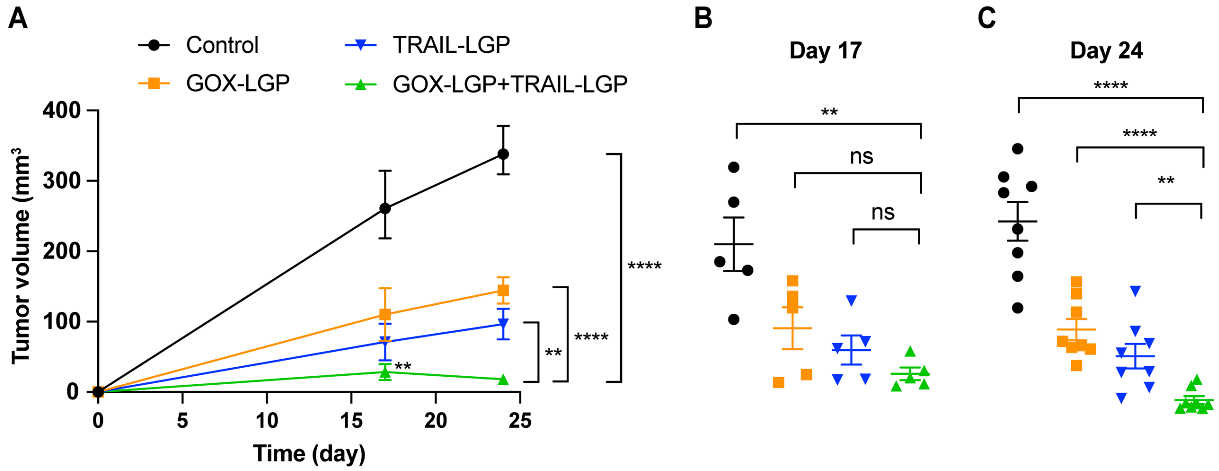

Figure S4. Tumor volume changes in response to TRAIL-LGP and GOx-LGP nanogels. (A) Tumor growth curve of murine HCC model after treatment of different groups (three or six intravenous doses of either GOx-LGP (0.4 mg/kg), TRAIL-LGP (3.5 mg/kg), or both in combination at 2–3-day intervals) (B) Tumor volume of HCC tumor after three intravenous doses of treatment (at D17 post-implantation) (n=5). (C) Tumor volume of HCC tumor after six intravenous doses of treatment (at D24 post-implantation) (n=8). Data are the mean values ± S.E.M., **p<0.01, ***p<0.001, ****p<0.0001.

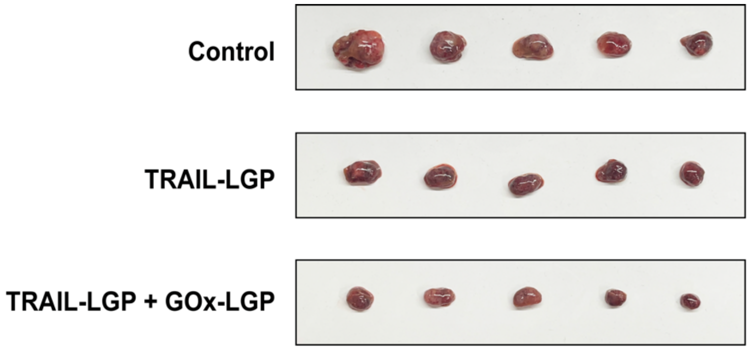

Figure S5. Representative images of tumor from orthotopic human JHH7 xenograft mice in response to either GOx-LGP (0.4 mg/kg), TRAIL-LGP (2.5 mg/kg), or both in combination at 2–3-day intervals (n=5).

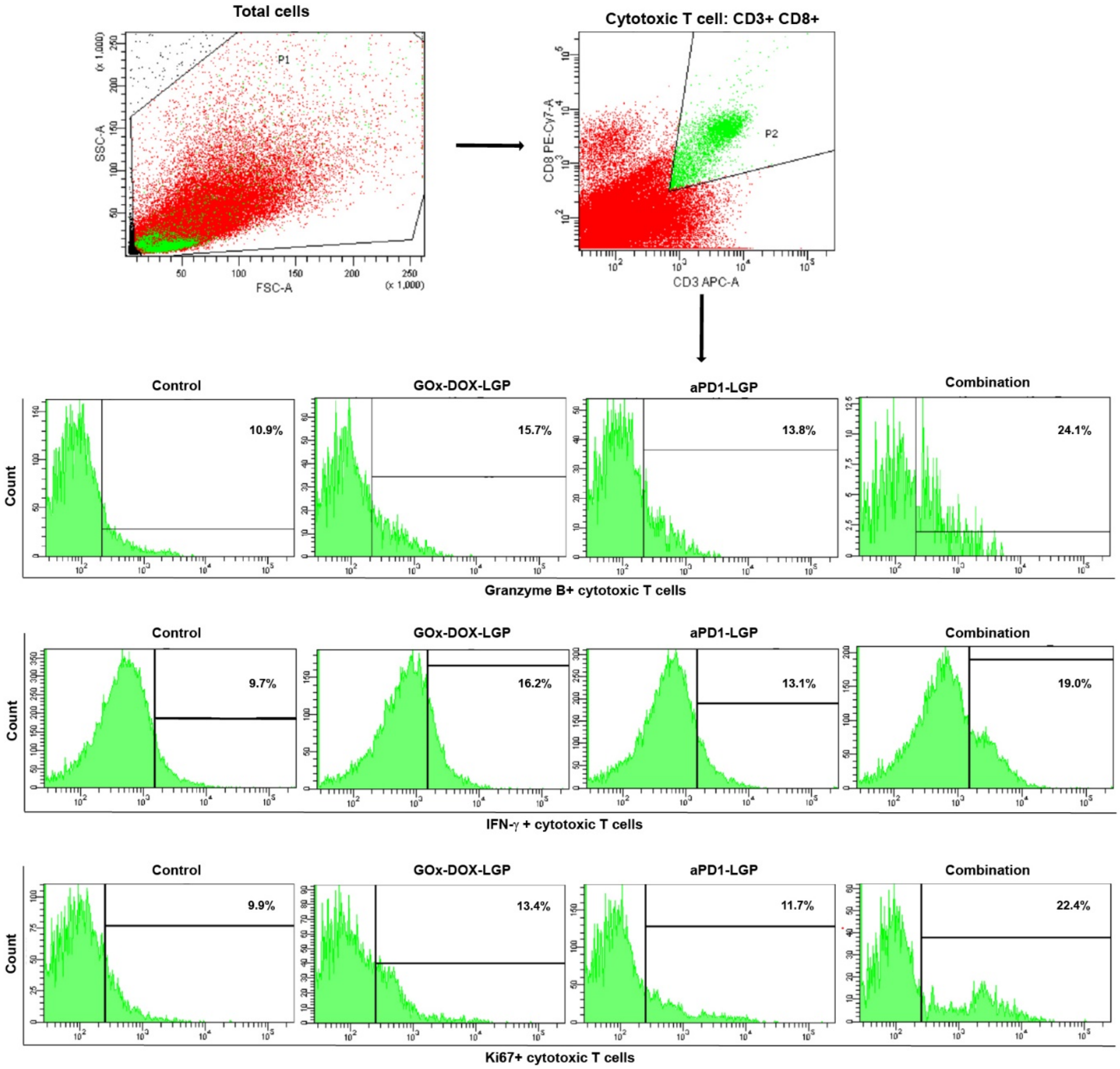

Figure S6. Gating strategy for flow cytometry analysis of granzyme B^{+}, IFN-γ^{+}, and Ki67^{+} cytotoxic CD8^{+} T cells in orthotopic HCC tumors following treatment with different formulations. Representative plots from individual samples are shown.

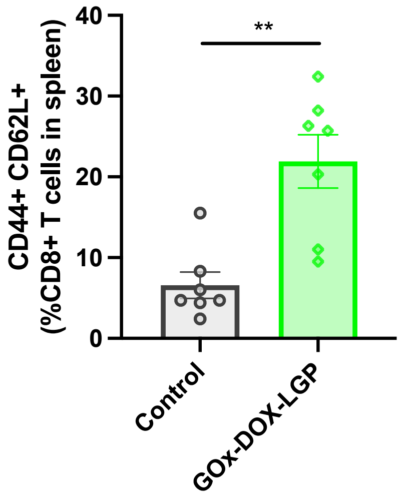

Figure S7. Treatment with GOx–DOX–LGP significantly increased the proportion of CD44^{+}CD62L^{+} central memory T cells in spleens of HCC tumor-bearing mice following treatment with GOx-DOX-LGP were detected by flow cytometry, indicating enhanced and long-term immune activation (n = 7). Data are the mean values ± S.E.M., **p<0.01.

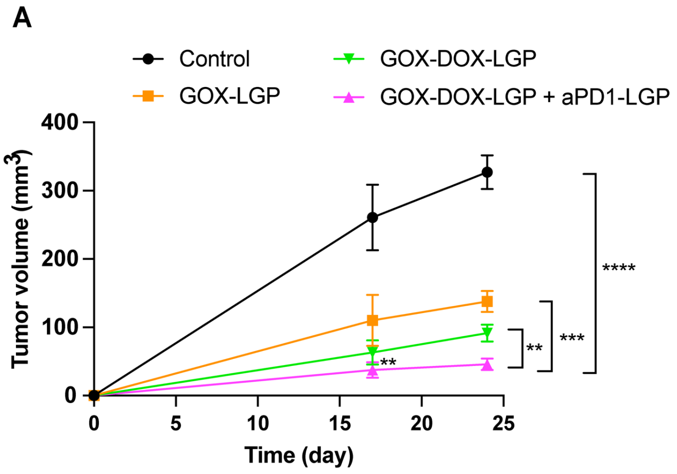

Figure S8. Tumor volume changes in response to Gox-DOX-LGP in combine with aPD1-LGP. (A) Tumor growth curve of murine HCC model after treatment of different groups (three or six intravenous doses of either GOx-DOX-LGP (GOx: ~0.4 mg/kg; DOX: ~1.8mg/kg) or aPD-1-LGP (1.4mg/kg) for single treatment groups, and both nanogels for combination treatment group at 2- to 3-d intervals. (B) Tumor volume of HCC tumor after three intravenous doses of treatment (at D17 post-implantation) (n=5); Data for the Control and GOx-LGP groups are shared with Fig. S4. (C) Tumor volume of HCC tumor after six intravenous doses of treatment (at D24 post-implantation) (n=8). Data are the mean values ± S.E.M., $^{**}p<0.01$, $^{***}p<0.001$, $^{****}p<0.0001$.

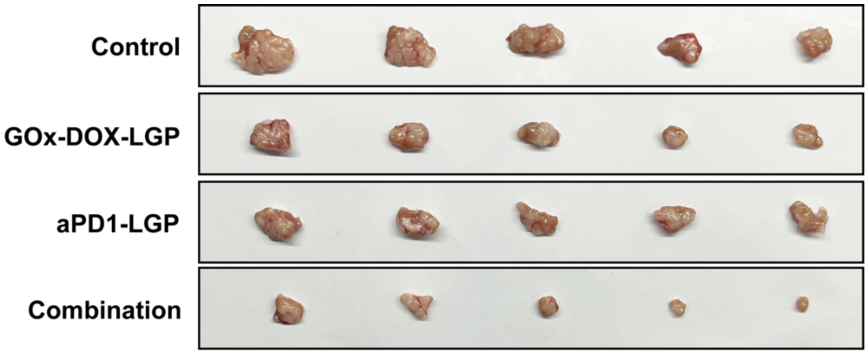

Figure S9. Representative images of murine orthotopic HCC tumors in response to either GOx-DOX-LGP (GOx: ~0.4 mg/kg; DOX: ~1.8mg/kg) or aPD-1-LGP (1.4mg/kg) for single treatment groups, and both nanogels for combination treatment group six times (at 2- to 3-d intervals) (n=5).

Reference

(1) Huang, H. C.; Sung, Y. C.; Li, C. P.; Wan, D.; Chao, P. H.; Tseng, Y. T.; Liao, B. W.; Cheng, H. T.; Hsu, F. F.; Huang, C. C.; *et al.* Reversal of pancreatic desmoplasia by a tumour stroma-targeted nitric oxide nanogel overcomes TRAIL resistance in pancreatic tumours. *Gut* **2022**, *71*, 1843–1855.

(2) Duarte, D.; Vale, N. Evaluation of synergism in drug combinations and reference models for future orientations in oncology. *Curr Res Pharmacol Drug Discov* **2022**, *3*, 100110.

(3) Anders, S.; McCarthy, D. J.; Chen, Y.; Okoniewski, M.; Smyth, G. K.; Huber, W.; Robinson, M. D. Count-based differential expression analysis of RNA sequencing data using R and Bioconductor. *Nat Protoc* **2013**, *8*, 1765–1786.

(4) Love, M. I.; Huber, W.; Anders, S. Moderated estimation of fold change and dispersion for RNA-seq data with DESeq2. *Genome Biol* **2014**, *15*, 550.

(5) Metsalu, T.; Vilo, J. ClustVis: a web tool for visualizing clustering of multivariate data using Principal Component Analysis and heatmap. *Nucleic Acids Res* **2015**, *43*, W566–570.

(6) Subramanian, A.; Tamayo, P.; Mootha, V. K.; Mukherjee, S.; Ebert, B. L.; Gillette, M. A.; Paulovich, A.; Pomeroy, S. L.; Golub, T. R.; Lander, E. S.; et al. Gene set enrichment analysis: a knowledge-based approach for interpreting genome-wide expression profiles. *Proc Natl Acad Sci U S A* **2005**, *102*, 15545–15550.
